# The PPAR α/γ Agonist Saroglitazar Improves Insulin Resistance and Steatohepatitis in a Diet Induced Animal Model of Nonalcoholic Fatty Liver Disease

**DOI:** 10.1038/s41598-020-66458-z

**Published:** 2020-06-09

**Authors:** Divya P. Kumar, Rebecca Caffrey, Jonathon Marioneaux, Prasanna K. Santhekadur, Madhavi Bhat, Cristina Alonso, Srinivas V. Koduru, Binu Philip, Mukul R. Jain, Suresh R. Giri, Pierre Bedossa, Arun J. Sanyal

**Affiliations:** 1 Department of Biochemistry, CEMR, JSS Medical College, JSS Academy of Higher Education and Research, Mysore, Karnataka India; 2 Sanyal Biotechnology LLC, Norfolk, Virginia USA; 3 OWL Metabolomics, Derio, Bizkaia, Spain; 4 Gene Arrays, Entity of Vedic Research, Inc, New York, USA; 5 Zydus Research Centre, Cadila Healthcare Limited, Ahmedabad, Gujarat India; 6 Department of Pathology, Hospital Beaujon, University Paris-Diderot, Paris, France; 7 Division of Gastroenterology, Hepatology and Nutrition, Virginia Commonwealth University, Richmond, Virginia USA

**Keywords:** Gastroenterology, Health care, Medical research

## Abstract

Insulin resistance and hepatic lipid accumulation constitute the metabolic underpinning of nonalcoholic steatohepatitis (NASH). We tested the hypothesis that saroglitazar, a PPAR α/γ agonist would improve NASH in the diet-induced animal model of NAFLD. Mice received chow diet and normal water (CDNW) or high fat western diet and ad lib sugar water (WDSW). After 12 weeks, WDSW fed mice were randomized to receive (1) WDSW alone, (2) WDSW + vehicle, (3) WDSW + pioglitazone or (4) WDSW + saroglitazar for an additional 12 weeks. Compared to mice on WDSW and vehicle controls, mice receiving WDSW + saroglitazar had lower weight, lower HOMA-IR, triglycerides, total cholesterol, and ALT. Saroglitazar improved steatosis, lobular inflammation, hepatocellular ballooning and fibrosis stage. NASH resolved in all mice receiving saroglitazar. These effects were at par with or superior to pioglitazone. Molecular analyses confirmed target engagement and reduced oxidative stress, unfolded protein response and fibrogenic signaling. Transcriptomic analysis further confirmed increased PPAR-target expression and an anti-inflammatory effect with saroglitazar. Lipidomic analyses demonstrated that saroglitazar also reduced triglycerides, diglycerides, sphingomyelins and ceramides. These preclinical data provide a strong rationale for developing saroglitazar for the treatment of NASH in humans.

## Introduction

Nonalcoholic fatty liver disease (NAFLD) encompasses a continuum of liver disease ranging from fatty liver (NAFL) to steatohepatitis (NASH), fibrosis and cirrhosis^1–3^. This rising prevalence of NASH is accompanied with an alarming increase in the number of patients with cirrhosis and hepatocellular carcinoma (HCC) necessitating liver transplantation^4,5^. Dynamic models of disease progression predict a doubling of the burden of end-stage liver disease from the NAFLD epidemic by 2030 if left unmanaged^6^. Despite progress in understanding the clinical drivers of disease progression and pathogenesis of NAFLD and an exponential increase in clinical trials investigating the therapeutic potential and identifying therapeutic targets, there are immediate unmet medical needs and challenges and the disease still remains without any approved drugs^7,8^.

A key consideration in therapeutic development for NASH is the identification of a rational therapeutic target. NASH often develops in the context of excess adiposity and systemic insulin resistance^9^. The current paradigm for the pathogenesis of NASH starts with increased delivery of lipids such as free fatty acids (FFA), carbohydrates along with inflammatory cytokines and gut-microbiome-derived products e.g. endotoxin^10^. This overloads the hepatocellular metabolic machinery resulting in accumulation of lipids, mainly triglycerides, and induction of cell stress which can trigger apoptotic and inflammatory signaling. Inflammation drives fibrogenic remodeling towards cirrhosis. This four-step paradigm has led to therapeutic approaches targeting either the upstream drivers such as insulin resistance or attempts to stop the downstream inflammatory-fibrogenic signals^11–13^. To date, the best results have been obtained with either agents with pleiotropic effects such as obeticholic acid or those that target upstream drivers like adiposity e.g. bariatric surgery^14^.

Peroxisome proliferation activating receptors (PPAR) are a part of a superfamily of nuclear hormone receptors and can be classified as PPAR-α, PPAR-β/δ and PPAR-γ. Pioglitazone is a PPAR-γ agonist and improves NASH histology^15,16^. Its utility is however limited by lack of universal benefit and development of weight gain^17^. PPAR-α agonists induce hepatic fatty acid oxidation and are expected to also reduce the metabolic overload leading to NASH. However, clinical trials with fibrates have been disappointing^18–20^. These have led to attempts to develop drugs that are selective (such as Pemafibrate, a novel selective peroxisome proliferator-activated receptor α modulator) or target more than one type of PPAR receptor to develop synergy against NASH^21–23^; Elafibrinor a PPAR-α/δ agonist is currently in pivotal trials for NASH (NCT02704403).

Saroglitazar is a PPAR-α/γ agonist. The rationale for its use in NASH includes the expected synergy of improved insulin resistance and increased lipid oxidation from its PPAR-γ and PPAR-α effects respectively, which would reduce the lipotoxic load to the liver^24^. It improves dyslipidemia associated with type 2 diabetes and is approved for this indication in India and no safety concerns currently exist with this molecule^25^. While it has been shown to improve steatosis, inflammation, ballooning and prevented development of fibrosis in mice with choline-deficient high-fat diet-induced NASH model^24^, its effects on NASH have not yet been fully evaluated in a relevant NASH model.

We here report the effects of saroglitazar on the development of NASH in the diet-induced animal model of NAFLD (DIAMOND) that recapitulates many of the key elements of human disease especially the histological progression and molecular signature of NASH^26–28^. The goals were to demonstrate: (1) efficacy against NASH, (2) impact on the systemic obese, inflammatory state, (3) effects on key pathogenic signaling pathways, metabolome and transcriptome involved in disease pathogenesis.

## Results

### Saroglitazar prevented diet-induced obesity and insulin resistance

**Figure 1:**
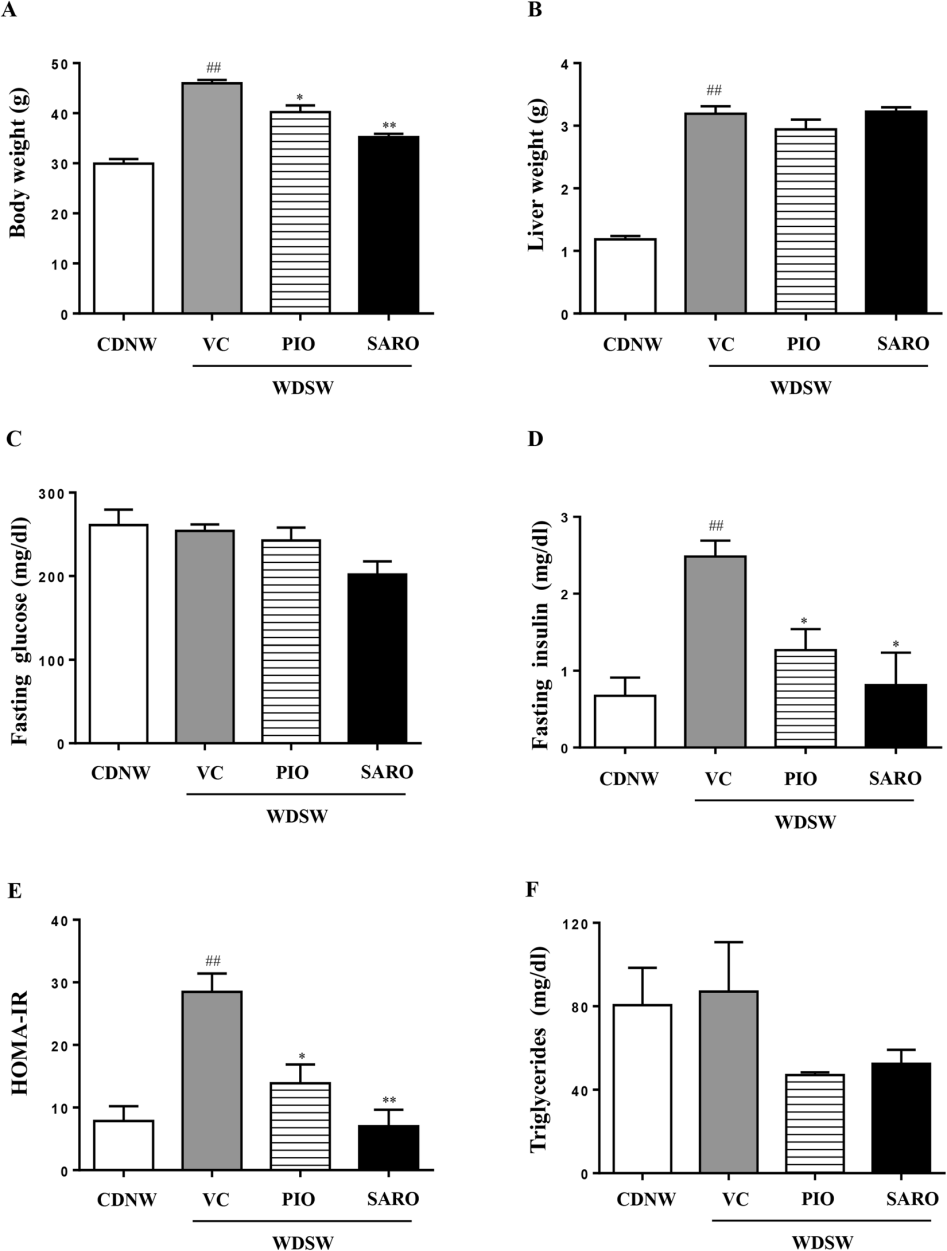
Preventive effect of saroglitazar on diet-induced obesity in DIAMOND mice. DIAMOND mice (B6/129 mice) were fed chow diet (CDNW) or high fructose/glucose, high fat western diet (WDSW) for up to 12 weeks to develop fatty liver and steatohepatitis. Mice were administered pioglitazone, saroglitazar or vehicle control along with CDNW or WDSW for an additional 12 weeks. (**A**) Body weight, (**B**) liver weight, (**C**) fasting glucose, (**D**) fasting insulin, (**E**) HOMA-IR, (**F**) serum triglycerides. Data are expressed as the mean ± SEM for 6–12 mice per group. ^##^p < 0.001 compared to CDNW; *p < 0.05, **p < 0.001 compared to WDSW, vehicle control.

DIAMOND mice fed a high fat diet with ad lib administration of sugar in drinking water (WDSW) developed rapid weight gain and increase in liver weight compared to mice fed a chow diet and normal water (CDNW) (Fig. 1A,B). In addition to developing obesity, feeding a WDSW diet also led to the development of insulin resistance (Fig. 1C–F). Data for mice fed WDSW alone or with vehicle control were similar and vehicle control data is used for statistical analyses and are shown as vehicle control (VC) group under WDSW in the figures. The mice treated with saroglitazar for 12 weeks showed significant reduction in the body weight compared to vehicle controls (Fig. 1A). The liver weight did not however change significantly (Fig. 1B). Saroglitazar also reduced fasting insulin levels in DIAMOND mice (Fig. 1D) and was reflected in a significant decrease in insulin resistance as measured by HOMA-IR (homeostatic model Assessment for insulin resistance) (Fig. 1E). The degree of improvement was comparable to that seen with pioglitazone, the positive control group.

### Saroglitazar decreased liver injury and dyslipidemia

**Figure 2:**
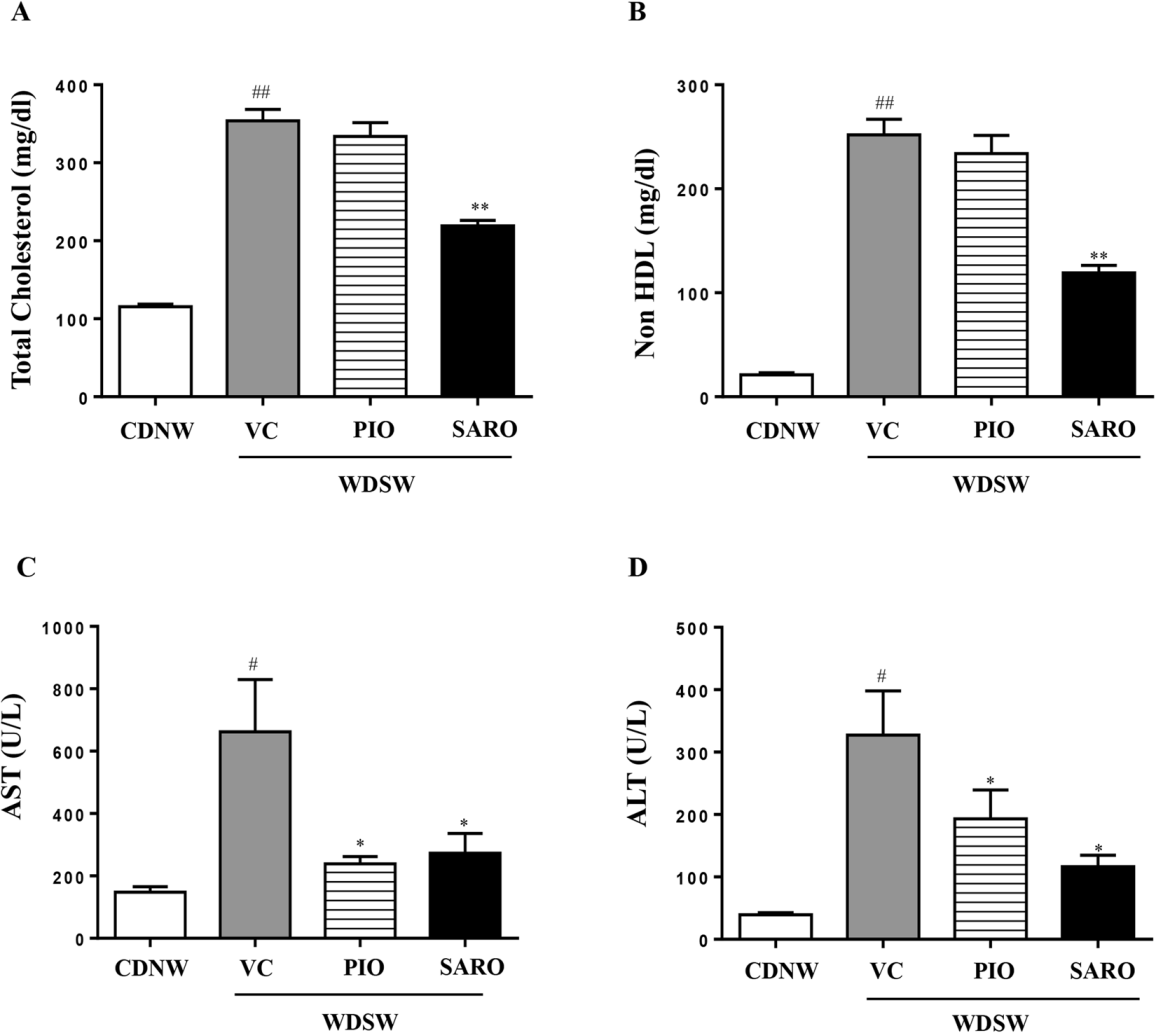
Saroglitazar decreased liver injury and dyslipidemia. DIAMOND mice fed CDNW or WDSW for 12 weeks were administered pioglitazone, saroglitazar or vehicle control for another 12 weeks. At the end of 24 weeks following dietary intervention and treatment, mice were fasted overnight and blood was collected. (**A**) serum cholesterol, (**B**) serum non HDL, (**C**) serum AST, (**D**) serum ALT. Data are expressed as the mean ± SEM for 6–12 mice per group. ^#^p < 0.05, ^##^p < 0.001 compared to CDNW; *p < 0.05, **p < 0.001 compared to WDSW, vehicle control. HDL, high density lipoprotein; AST, aspartate aminotransferase; ALT, alanine aminotransferase.

Serum total cholesterol, non-HDL cholesterol, AST and ALT increased in WDSW group compared to chow-fed mice (Fig. 2A–D). Pioglitazone reduced circulating triglycerides without affecting total of non-HDL cholesterol. Saroglitazar improved both circulating triglycerides and cholesterol parameters compared to WDSW with or without vehicle control groups (Figs. 1F and 2A–D, p < 0.001- total cholesterol, non HDL; p < 0.05- AST and ALT). WDSW controls had increased levels of serum AST and ALT reflective of liver injury. These were reduced significantly by both pioglitazone and by saroglitazar to comparable levels. Saroglitazar also increased circulating adiponectin and decreased TNF-α values compared to WDSW VC controls (Supplementary Fig. 2A,B).

Collectively, these data demonstrates that diet-induced obesity in DIAMOND mice is significantly attenuated by the treatment of saroglitazar. The degree of improvement was at par with pioglitazone with the exception of cholesterol-related parameters.

### Saroglitazar ameliorates fatty liver, steatohepatitis and fibrosis

**Figure 3:**
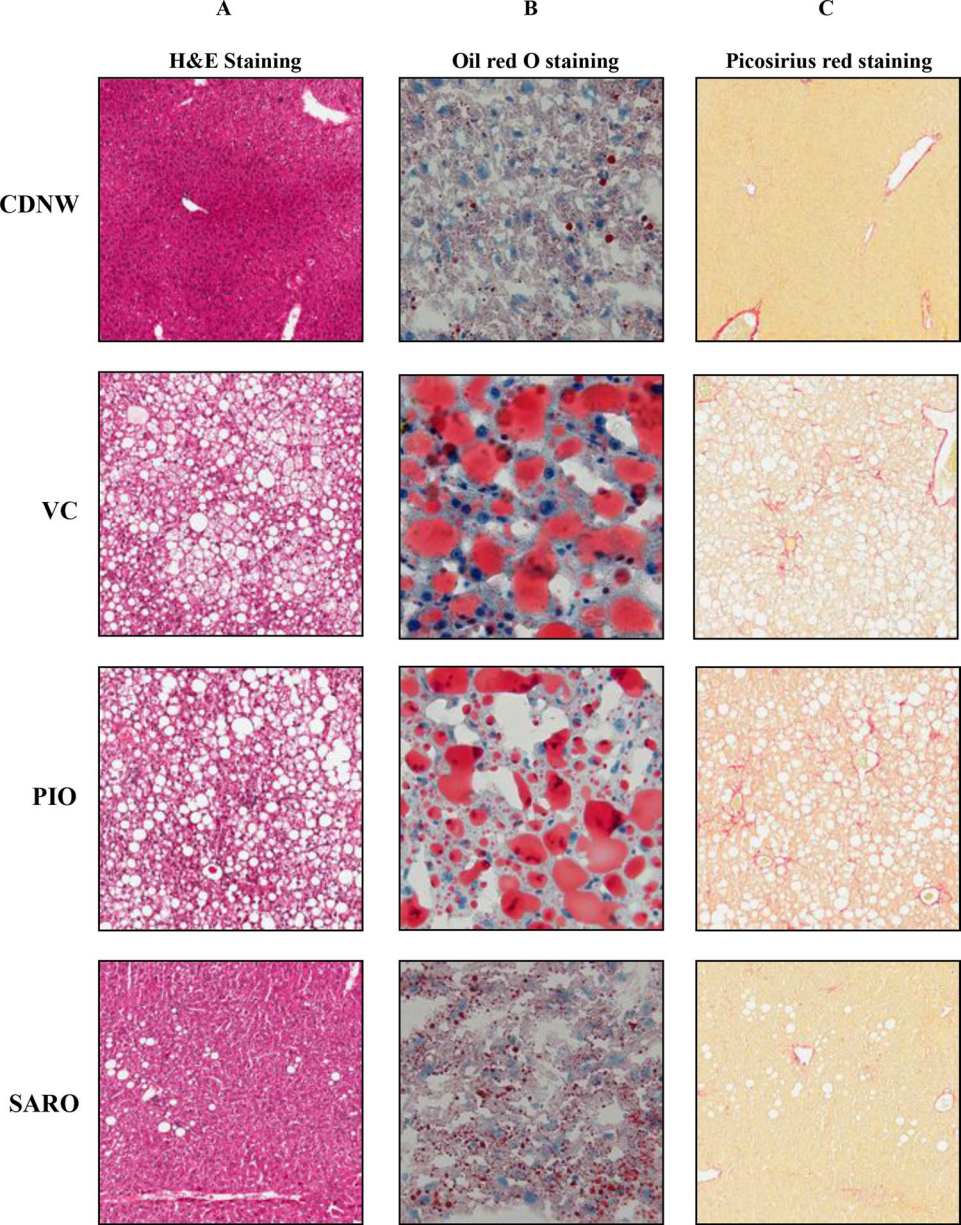
Saroglitazar treatment improved the liver histology of NASH in DIAMOND mice. DIAMOND mice were treated with pioglitazone, saroglitazar or vehicle control for 12 weeks along with CDNW or WDSW, which had developed fatty liver and steatohepatitis. Representative liver sections stained with Hematoxylin and Eosin (H&E), Picosirius red and Oil Red O.

**Figure 4:**
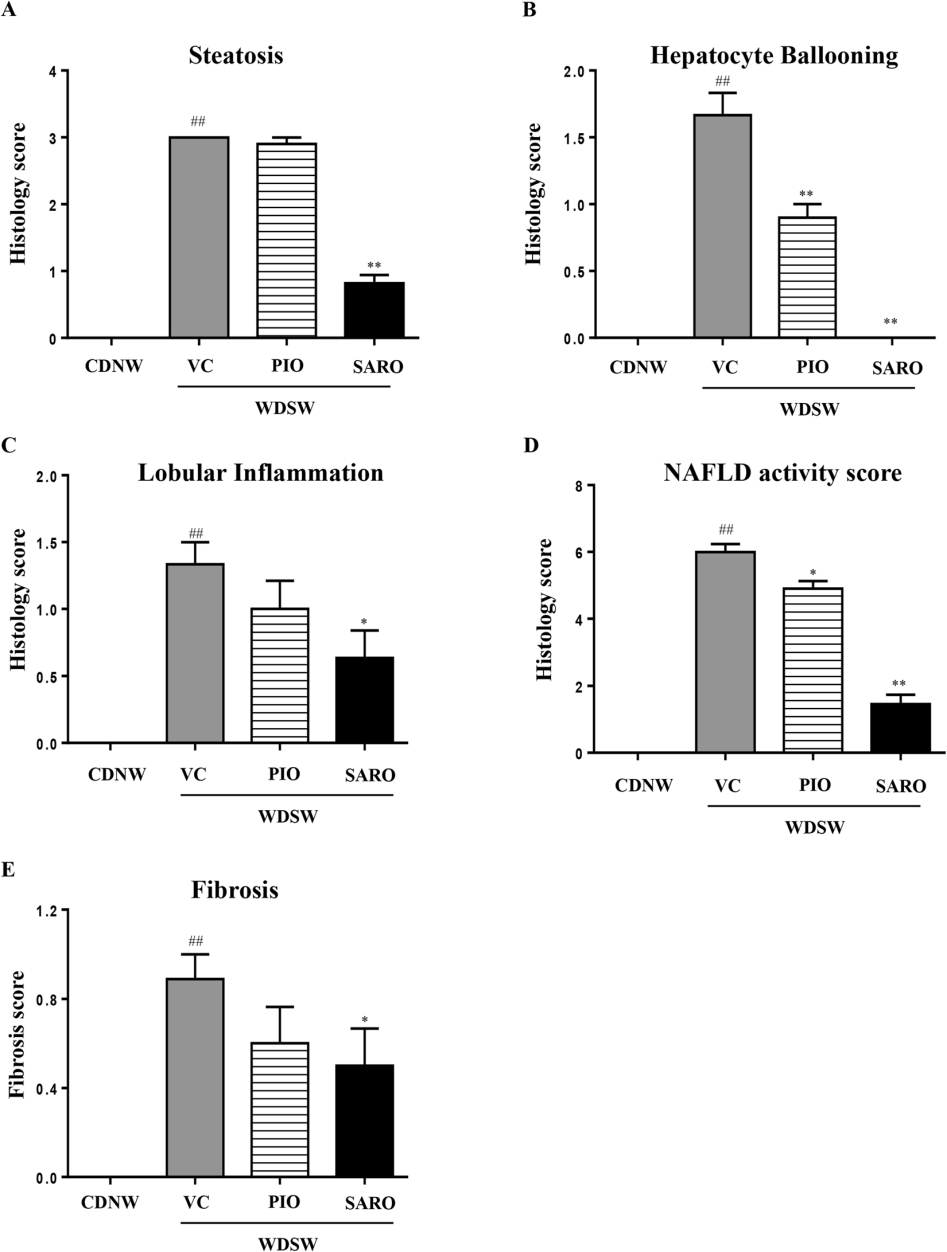
Saroglitazar treatment ameliorates fatty liver, steatohepatitis and fibrosis. DIAMOND mice were treated with pioglitazone, saroglitazar or vehicle control for 12 weeks along with CDNW or WDSW. Mice were maintained on this diet for 12 weeks prior to the initiation of treatment to develop fatty liver and steatohepatitis. Histology score for (**A**) steatosis, (**B**) hepatocyte ballooning, (**C**) lobular inflammation, (**D**) NAFLD activity score and (**E**) fibrosis were quantified. Data are expressed as the mean ± SEM for 6–12 mice per group. ^##^p < 0.001 compared to CDNW; *p < 0.05, **p < 0.001 compared to WDSW, vehicle control.

Mice fed CDNW had normal liver architecture and histology (Fig. 3, top row). In contrast, mice on WDSW with vehicle control developed grade 3 macro vesicular steatosis along with some micro vesicular steatosis as have been described before (Fig. 3, second row). Steatosis was confirmed by Oil-Red-O staining as well (Fig. 3 middle column). This was accompanied by a mean hepatocellular ballooning score of 1.7 (±0.5) and lobular inflammation score of 1.3 (±0.7) (Fig. 4A–C). Together, these resulted in a mean (±S.D.) NAS of 6 (±0.7) and SAF activity score of 3 (±0.7). All of the WDSW mice had steatohepatitis.

Pioglitazone reduced the mean NAS to 4.9 (±0.7) and SAF activity to 2 (±0.6). Of note, even though pioglitazone reduced ballooning mainly, only 2 of 10 mice were considered not to have steatohepatitis based on an “overall” gestalt diagnosis by the pathologist. In contrast, saroglitazar significantly reduced steatosis, hepatocellular ballooning, and lobular inflammation (p < 0.001 for all). Ballooning was absent in all saroglitazar treated mice and none of the mice were considered to have steatohepatitis; 9/12 mice had residual fatty liver and 3/12 had no histological evidence of NAFLD. The overall NAS was only 1.45 (±0.9) in the saroglitazar treated mice and significantly lower than that in the WDSW vehicle group (Fig. 4D). The mean fibrosis stage in WDSW vehicle controls was 0.7 (±0.4). Pioglitazone reduced the mean fibrosis stage to 0.6 and saroglitazar reduced the mean fibrosis stage to 0.54 (p < 0.05 vs. VC for saroglitazar only, Fig. 4E). These data-trends were further confirmed by collagen morphometry but did not reach significance (Supplementary Fig. 4C). Overall, histologically the effects of saroglitazar were superior to pioglitazone.

### Saroglitazar reduces hepatic ER stress, inflammation and fibrogenic signaling

**Figure 5:**
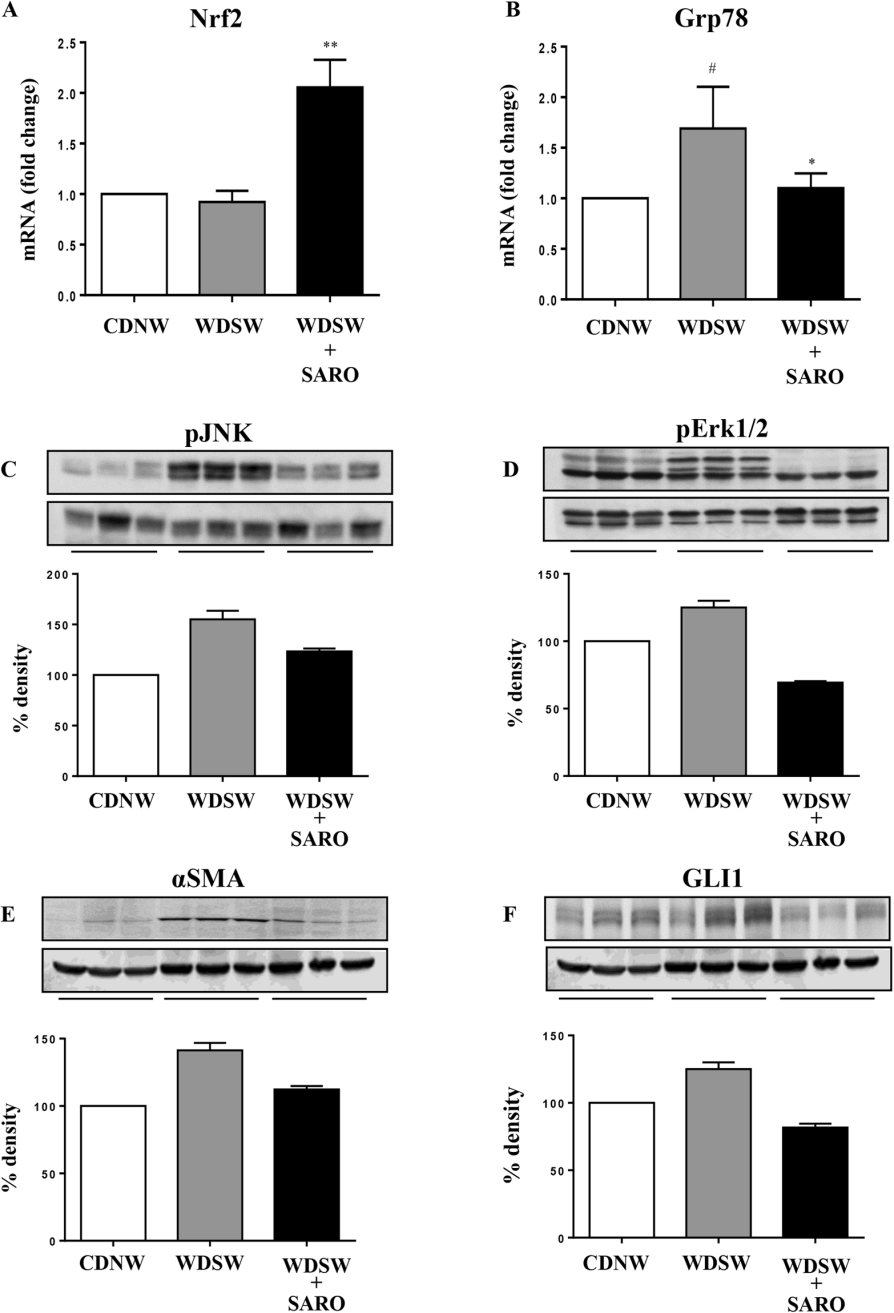
Saroglitazar reduces hepatic ER stress, inflammation and fibrogenic signaling. The relative expression of hepatic mRNA levels of Nrf2 (**A**) and Grp78 (**B**) were determined using TaqMan Q-PCR. The experiments were carried out in triplicates and β-actin was used as endogenous control for normalizing the mRNA levels. Whole cell lysates were prepared from liver tissue of DIAMOND mice treated with saroglitazar or vehicle control for 12 weeks along with CDNW or WDSW, which had developed fatty liver and steatohepatitis. Immunoblot analyses were performed for p-JNK (**C**), p-Erk1/2 (**D**), αSMA (**E**), GLI1 (**F**). Bar graphs show the densitometric values calculated after normalization to loading control, β-actin. Data are expressed as the mean ± SEM for 6–12 mice per group. ^#^p < 0.05 compared to CDNW; **p < 0.001 compared to WDSW, vehicle control. nrf2, nuclear factor erythroid 2-related factor 2; Grp78, glucose regulated protein 78; JNK, c-Jun N-terminal kinases; Erk1/2, extracellular signal regulated protein kinases 1 and 2; αSMA, alpha smooth muscle actin; GLI1, glioma-associated oncogene homolog 1. Unprocessed original scans of the western blots in C-F are shown in Supplementary Fig. 9.

Increased oxidative stress and unfolded protein response are key downstream mechanisms of tissue injury and inflammation in NASH^29,30^. The transcriptional factor nrf2 protects against oxidative stress^31,32^. Saroglitazar led to a significant upregulation of Nrf2 gene expression compared to vehicle controls on WDSW (Fig. 5A). It was also associated with increased superoxide dismutase expression (Supplementary Fig. 3A). The activation of the unfolded protein response was noted in WDSW fed vehicle controls by an increase in Grp78^27,33^; saroglitazar reduced Grp78 expression to levels seen with chow diet (Fig. 5B). The expression of inflammatory markers such as TNF-α and IL-1β were also reduced in the saroglitazar treated mice (Supplementary Fig. 3B,C). The downstream activation of inflammatory pathways JNK and ERK were also noted in WDSW fed vehicle controls (Fig. 5C,D). Saroglitazar reduced the activation status of both pathways (P < 0.001) compared to vehicle controls. The activation of hepatic stellate cells was also decreased by saroglitazar as evidenced by decreased collagen and α-smooth muscle actin gene and protein expression (Fig. 5E and Supplementary Fig. 4A,B). The expression of the pro-inflammatory/fibrogenic Hedgehog signaling mediator GLI1 was also decreased by saroglitazar compared to the WDSW fed vehicle control group (Fig. 5F).

### Saroglitazar activates PPAR-α and PPAR-γ targets

To determine target engagement, the expression of key PPAR-α and PPAR-γ targets were measured. Compared to mice fed WDSW and vehicle, mice receiving WDSW + saroglitazar had upregulation of ACOX1, CPT1A and LPIN2 (PPAR alpha target genes- Supplementary Fig. 5A–C) and UCP2 and CD36 (PPAR gamma target genes- Supplementary Fig. 5D,E).

### The transcriptomic signature of saroglitazar in NASH reflects predominantly metabolic and immune-inflammatory effects on NASH

**Figure 6:**
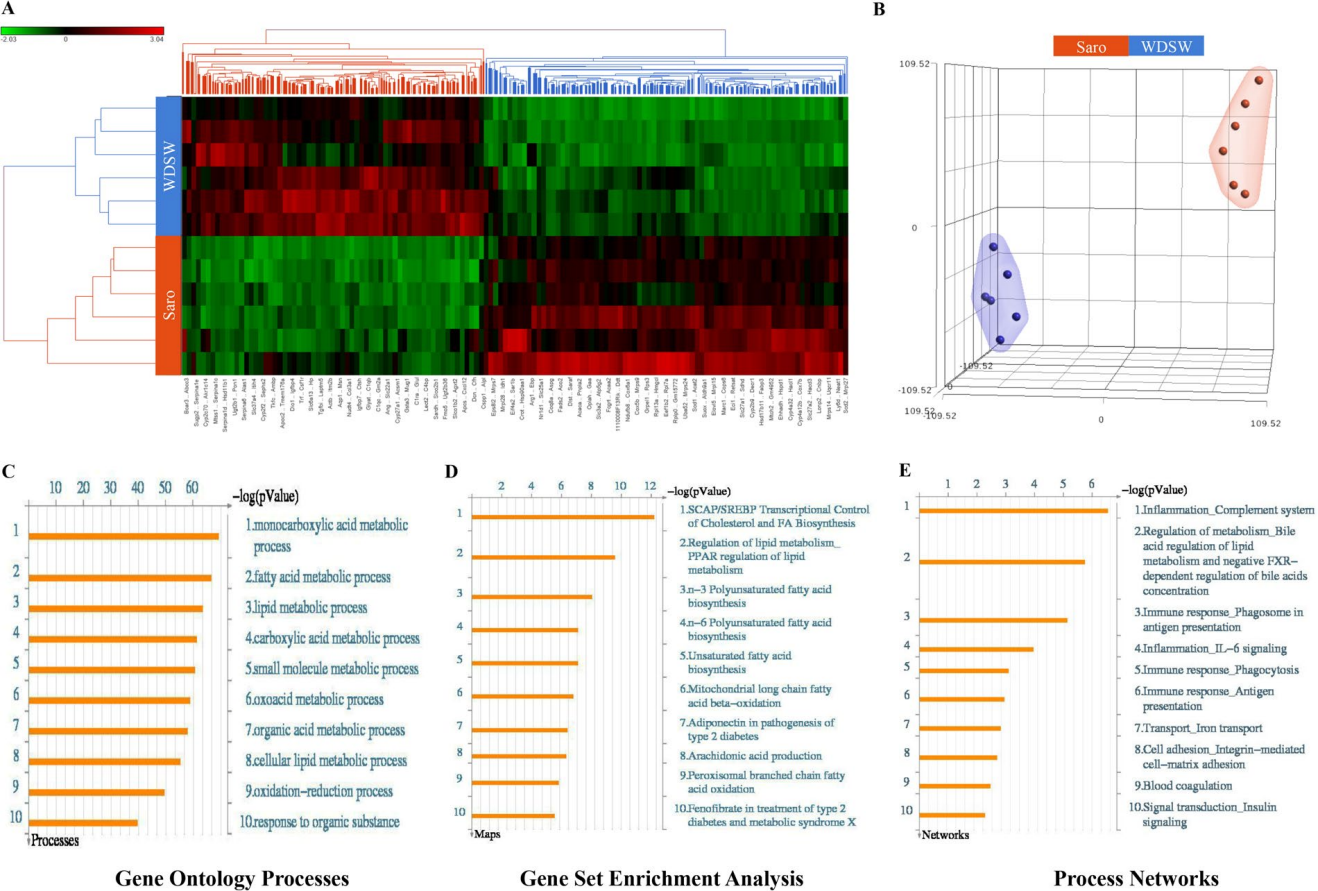
Hepatic gene expression data set in saroglitazar treated DIAMOND mice. Transcriptomic analysis was performed on liver tissues from saroglitazar treated mice fed with CDNW or WDSW for 12 weeks. The data are presented as (**A**) heat map demonstrating differentially expressed genes. Red and green colors indicate high and low gene expression, respectively, (**B**) t-SNE plots showing well segregated saroglitazar and WDSW groups, (**C**) Gene ontology (GO) processes; (**D**) Gene set Enrichment Analysis (GSEA); and (**E**) Process Networks analysis in saroglitazar treated mice versus untreated mice on WDSW. The top rank ordered processes, maps and networks are based on statistical significance.

Heat map visualization of the hepatic transcriptome demonstrated distinct differences between WDSW fed vehicle controls and WDSW fed mice receiving saroglitazar (Fig. 6A,B). Principal component analysis plots further confirmed these findings. Differentially expressed mRNA were quantified (FDR < 0.05) with 262 mRNAs showing greater than 2 fold change, in which 134 genes were found to be upregulated and the remaining down regulated (Supplementary Table 1).

Both pathway enrichment and gene ontology enrichment (Fig. 6C,D) confirmed that the principal differentially expressed mRNA following saroglitazar treatment on the background of WDSW were involved in metabolic pathways. The top three pathways enriched included the SCAP/SREBP pathway, PPAR-signaling pathway and n3 polyunsaturated fatty acid biosynthesis pathway. It is notable that at a mRNA level, the expressions of several lipogenic genes were upregulated including fatty acid synthetase, elongase and steroyl coA desaturase. As expected, PPAR-alpha targets such as fatty acid binding protein, ACAA, CPT1b were all upregulated (Supplementary Fig. 6).

Several process networks were also modified by saroglitazar (Fig. 6E). Process networks refer to a pre-set network of protein interactions characteristic for the process. These indicated that in addition to metabolic pathways there was an enrichment of expression of genes involved in inflammation, immune regulation and bile acid metabolism. We further evaluated the interactions between networks that were seen with saroglitazar. This was done in an unbiased manner using the Analyze Networks (AN) algorithm with default settings. This is a variant of the shortest paths algorithm with main parameters of relative enrichment with the uploaded data, and relative saturation of networks with canonical pathways. The lead network included Legumain, HSP60, MHC class II, TRAF6, TAK1 (MAP3K7) which are linked to modulation of immune responses (Supplementary Fig. 7). Overall these indicate that in the setting of WDSW feeding, saroglitazar affected multiple metabolic pathways and genes related to immune activation, phagocytosis and inflammation.

### Saroglitazar reduces lipid load and lipotoxic compounds in the liver

**Figure 7:**
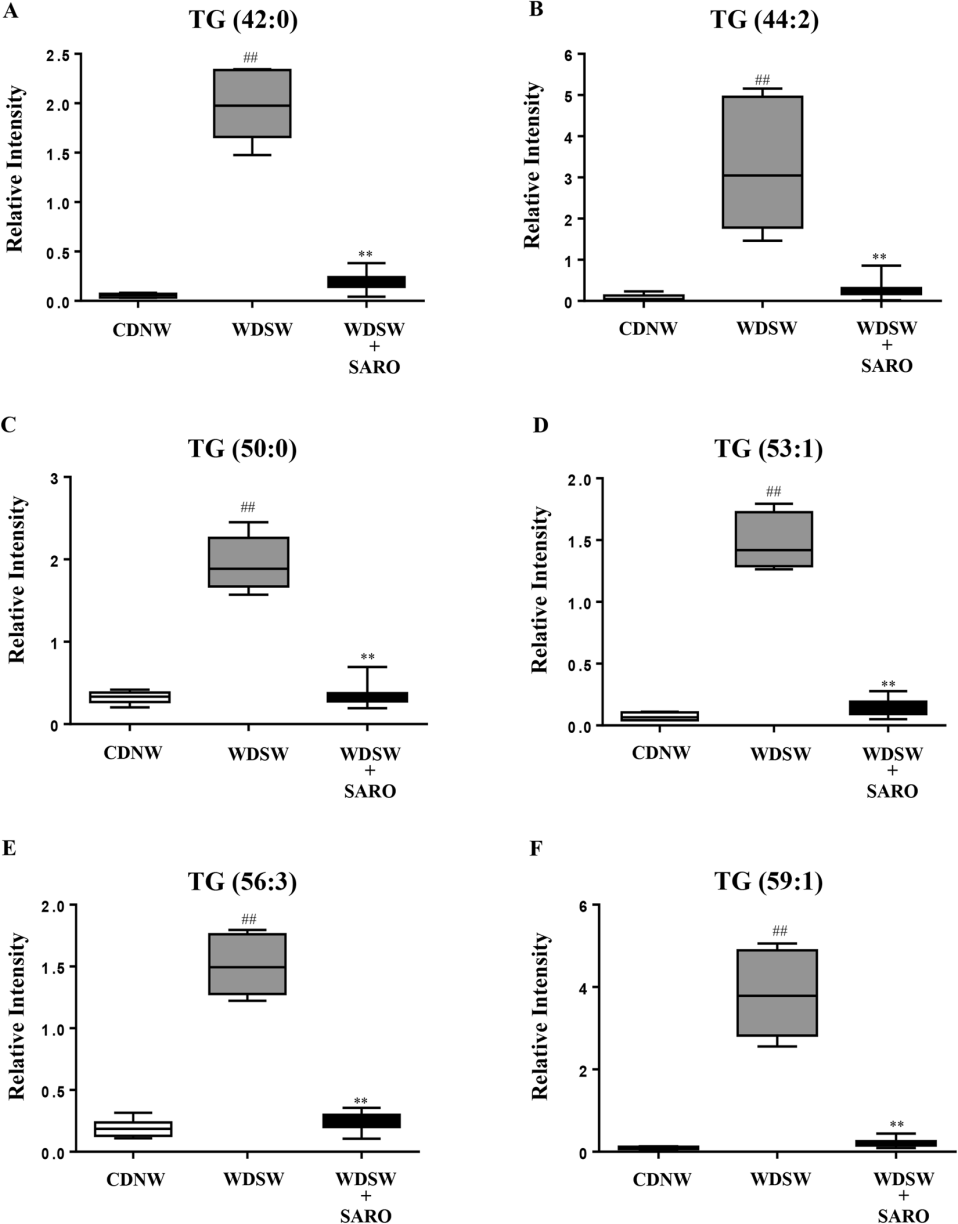
Saroglitazar treatment decreases triglyceride profile in DIAMOND mice. Triglyceride (TG) profiling was performed in the liver samples of mice treated/untreated with saroglitazar fed on CDNW or WDSW using ultra high performance liquid chromatography- mass spectrometry (UHPLC-MS). Box plots of (**A**) TG (42:0), (**B**) TG (44:2), (**C**) TG (50:0), (**D**) TG (53:1), (**E**) TG (56:3), and (**F**) TG (59:1). Data are expressed as the mean ± SEM for 6–12 mice per group. ^##^p < 0.001 compared to CDNW; **p < 0.001 compared to WDSW, vehicle control.

**Figure 8:**
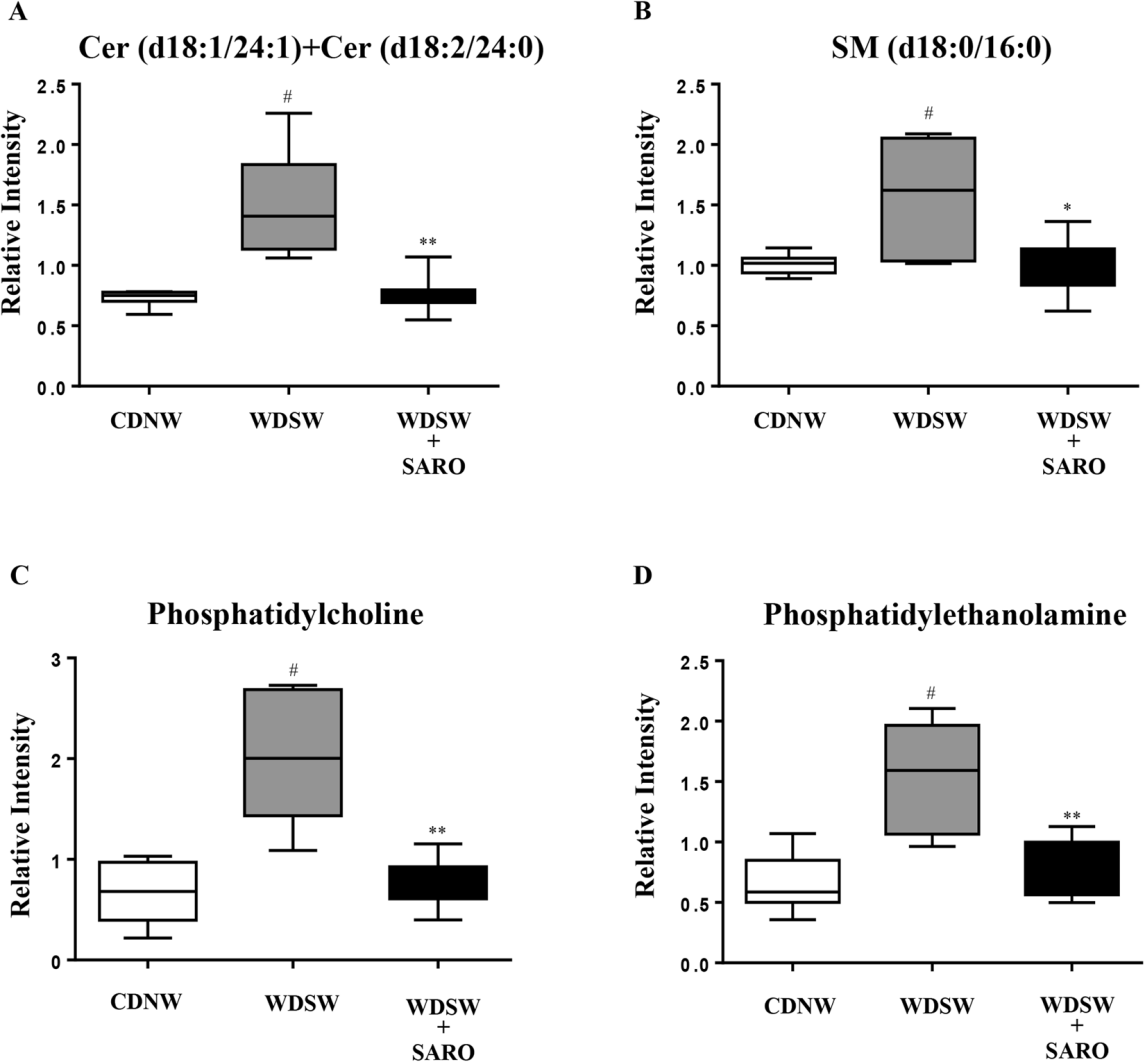
Saroglitazar treatment reduces lipotoxic compounds in the liver. Endogenous metabolic profiles of liver samples taken from mice fed with CDNW or WDSW treated with/without saroglitazar were determined using ultra high performance liquid chromatography- mass spectrometry (UHPLC-MS). Box plots of (**A**) ceramide, (**B**) sphingomyelin, (**C**) phosphatidylcholine, (**D**) phosphatidylethanolamine. Data are expressed as the mean ± SEM for 6–12 mice per group. ^#^p < 0.05, ^##^p < 0.001 compared to CDNW; *p < 0.05, **p < 0.001 compared to WDSW, vehicle control.

Metabolomic analyses demonstrated that saroglitazar produced a global decrease in triglyceride and diglyceride species (Fig. 7 and Supplementary Fig. 8A,B). Several species of triglycerides were reduced to levels seen in chow-diet fed mice. There was also a decrease in total ceramides, sphingomyelins, phosphatidylcholines, and phosphatidylethanolamine in mice receiving saroglitazar from the elevated levels noted in chow fed mice (Fig. 8A–D). Saroglitazar administration increased the levels of central carbon metabolism related metabolites, including carboxylic acids, nucleosides and nucleotides and redox electron carriers (Redox) (Supplementary Fig. 8C,D).

## Discussion

There remains an acute need for development of safe and effective drugs for the treatment of NASH. The current study evaluated the effects of saroglitazar, a PPAR-α/γ agonist in a preclinical model of NASH. It demonstrated a systemic effect of the drug with decreased weight gain, improved insulin sensitivity (HOMA-IR, adiponectin), dyslipidemia (triglycerides and cholesterol) along with resolution of steatohepatitis and improvement in all of the key histological features of NASH. This was accompanied by evidence of both PPAR-α (ACOX1, CPT1A and LPIN2) and PPAR-γ (CD36, UCP2) target engagement. Molecular analyses confirmed reduced cell stress, inflammatory and fibrogenic signaling. Unbiased transcriptomic analyses further confirmed a more global anti-inflammatory effect. Finally, lipidomic analyses confirmed a decrease in triglycerides along with reduced diacylglycerols and several lipotoxic species. These provide a rationale for the study of saroglitazar for the treatment of NASH in humans.

Drugs for NASH must be both safe and effective. So far, saroglitazar has been used in diabetic patients with dyslipidemia and there are no reports of toxicity^34–37^. In the current study also, mice tolerated the compound well. These are reassuring data to move saroglitazar into human trials. Of note, early phase 2A data presented at the annual meeting of the American Association for Study of Liver Diseases (AASLD) in 2019 also did not find any issues with tolerability.

It is interesting to note that saroglitazar had superior histological benefit compared to pioglitazone despite similar effects on insulin resistance markers such as HOMA-IR (Fig. 1). This is partly due to benefits of saroglitazar and the relative modest efficacy of pioglitazone in several mice models of NAFLD. Pioglitazone has two enantiomers the R and S enantiomers^38^. The R enantiomer is responsible for the hepatic insulin sensitizing effects. The hepatic bioavailability of these enantiomers varies across species^38,39^ and is the most likely explanation for a less than expected impact of pioglitazone. The principal focus of this study was to evaluate saroglitazar and the specific distribution of enantiomers of pioglitazone was beyond the focus and scope of the current manuscript. Further, pioglitazone was chosen as a control because it is a PPAR-γ agonist to serve as a positive control in case no effects were noted with saroglitazar and not necessarily to compare their relative efficacy. The value of pioglitazone is already established in humans^40,41^ and the utility of saroglitazar now awaits testing in human trials.

There is a strong signature for PPAR-α activation with increased fatty acid oxidation genes in line with the known predominantly PPAR-α agonistic properties of saroglitazar^34^. This indicates that fat oxidation is likely to be important for the observed effects. Surprisingly a number of lipogenic gene expression was increased. This occurred despite a decrease in plasma insulin and may reflect a response to increased fat oxidation. However, the overall lipidomic profiles along with histological data do not support de novo lipogenesis pathway increase. Also the improvement in insulin resistance would be expected to reduce the delivery of glucose to the liver for de novo lipogenesis. In future studies, functional assessments of these pathways are warranted to fully resolve these issues.

Another novel observation is the decrease in both ER stress markers and activation of JNK and ERK. Cholesterol, ceramides and other lipotoxic sphingolipids are known to induce cell stress and inflammation^42,43^. The observed decrease in these lipids provides a reasonable explanation for the observed decrease in markers of cell stress and inflammation. Reduced inflammation was further noted with unbiased analyses of the hepatic transcriptome in this model. Inflammation is also well known to drive fibrogenic signaling^44^ and reduced inflammation is the most likely cause of reduced fibrogenic signaling noted with this molecule.

It is well known that many factors limit translation of preclinical data to humans. The model chosen for this study has been validated to recapitulate the key elements of human disease^26,27^. It is however prudent to acknowledge that there may be a translatability gap with this model as with other models. Also, given the mainly metabolic mechanism of action of saroglitazar, the study was conducted mainly to assess the effects of the drug on steatohepatitis. Separate studies, beyond the scope of this paper, are needed to evaluate the effects of this drug when advanced fibrosis is already established. This usually takes 32–40 weeks to be established in this model.

As with most studies, this study has some limitations as well. The spectrum and drivers of disease vary between males and females^45,46^. In this study, we only studied male mice. Additional studies in mice and humans are needed to evaluate any response variations across genders. Also, due to the logistics of running multiple groups simultaneously, it was not feasible to do a dose-ranging study with saroglitazar. When this drug is given to humans, it will also be important to assess its dose-response characteristics to pick the right dose for advanced phase trials.

In summary, the current study demonstrates that saroglitazar can improve steatohepatitis and potentially impact early fibrosis in a preclinical model of NASH that mimics many aspects of human disease^26,27^. These data support further assessment of saroglitazar in humans for the treatment of NASH.

## Methods

### Animal model

DIAMOND mice, an isogenic mouse strain derived from C57BL/6J and 129S1/SvImJ background (B6/129) used in the study were maintained with inbreeding as described previously^26^. All mice were housed in a 12 hour light/dark cycle in a 21–24 °C animal facility administered by the Division of Animal Resources, Eastern Virginia Medical School. The mice were weighed, anesthetized and euthanized at varying time points following initiation of dietary intervention for blood collection and tissue harvesting after overnight fasting. In addition to liver, several organs were harvested and weighed accordingly. All procedures were performed according to the approved protocols and in accordance with the guidelines and regulations of the Institutional Animal Care and Use Committee of Eastern Virginia Medical School (IACUC 16-011-002 Zydus Cadila Saroglitazar-001).

### Study design and interventions

8 week old male mice were fed ad libitum a high fat diet, high carbohydrate diet (Western diet, WD) with 42% kcal from fat and containing 0.1% cholesterol (Harlan TD.88137) with a high fructose-glucose solution (SW, 23.1 g/L d-fructose + 18.9 g/L d-glucose), referred to as Western diet sugar water (WDSW), as previously described (25) for 12 weeks. Control mice were fed a standard chow diet (CD, Harlan TD.7012) with normal water (NW). The DIAMOND mice fed with WDSW were then randomly divided into four groups: WDSW alone, vehicle control group- VC (WDSW, Tween 80 and 0.5% Na-CMC in a ratio 0.5:99.5), pioglitazone group- PIO (WDSW + pioglitazone, 30 mg/kg/day) and saroglitazar group- SARO (WDSW + saroglitazar, 4 mg/kg/day). Pioglitazone served as a positive control. The vehicle control group served to ensure that any observed effects were not due to the vehicle by comparison to a group fed WDSW alone. The drugs were administered once daily by oral gavage for a period of 12 weeks (Supplementary Fig. 1). The mice were monitored and the body weight was recorded on a weekly basis. At the end of the treatment, mice were fasted; blood collected and biochemical analysis were performed (Supplementary methods).

### Choice of drug dosages

The dose of saroglitazar was based on the body surface area (BSA) conversion factor as recommended by the Food and Drug Administration of the US federal government^47^. Below is the detail discussion considering the 4 mg dose of saroglitazar multiplying human (mg/kg) dose with the body surface area (BSA) conversion factor for mice (12.3) and then again multiplying it with inter species conversion factor 5, a dose of 4 mg/kg was selected. The dose of pioglitazone was similarly calculated and is in line with the published literature.

### Liver histology analysis, quantitative real-time PCR, Western blots and ELISA

These were performed as previously described^26,28^. A detailed description is provided in supplementary methods.

### Transcriptomics

#### Library preparation and sequencing for mRNA

The cDNA libraries were prepared using the NEXTflex Illumina Rapid Directional RNA-Seq Library Prep Kit (BioO Scientific) as per the manufacturer’s instructions^48^. Briefly, mRNA fraction was reverse transcribed, followed by PCR amplification and SPRI bead purification (Beckman Coulter). The unique index sequences were incorporated in the adaptors for multiplexed high-throughput sequencing and final library was assessed for size distribution and concentration using BioAnalyzer High Sensitivity DNA Kit (Agilent Technologies). Pooled libraries were sequenced on HiSeq 2500 according to the manufacturer’s instructions^49^.

#### Data processing

De-multiplexed and adapter-trimmed sequencing reads were generated using Illumina bcl2fastq (released version 2.18.0.12) allowing no mismatches in the index read. BBDuk was used to further trim the adapter sequences using “ktrim = r k = 23 mink = 11 hdist = 1” option.

### Analysis of transcriptomic data

#### mRNA sequencing data alignment and annotations

mRNA sequence data were uploaded to a High Performance Computing system by PartekFlow software (v7.0, Partek, St. Louis, MO), adapter-trimmed and remapped to mouse genome, mm10 using STAR v2.5.3a aligner with default setting (phred: 20) for read mapping. mRNA reads were annotated to RefSeq v84. Expression matrices were compared saroglitazar vs. WDSW. Statistical analyses were carried out using false discovery rate (FDR) correction through the Benjamin-Hochberg method. A default FDR < 0.05 was considered statistically significant^50,51^ with a log_2_-fold change more than 1 (Total count >50, p value < 0.05, FDR < 0.05; low expressed: 100% of samples have < = 100 reads).

#### Biological processes and gene network visualization by MetaCore

Biological pathway interactions of mRNA expressions were analyzed using MetaCore pathway analysis of differentially expressed genes (Clarivate Analytics, New York, NY) with *p* < 0.05 and greater than two-fold change. We performed enrichment analysis on saroglitazar vs. WDSW group. Functional gene networks were built based on differentially regulated mRNA gene lists as input to generate disease biomarkers and Gene Ontology terms (26,51).

### Metabolic profiling

Frozen liver samples, 6–12 from each group were analyzed by a combined metabolomics/lipidomics approach with dedicated platforms to extract and measure, among others, (1) fatty acyls, bile acids, steroids, and lysoglycerophospholipids; (2) glycerolipids, glycerophospholipids, sterol lipids, and sphingolipids; (3) amino acids and derivatives; and (4) polar metabolites. This combined analysis was established for rodent liver by OWL Metabolomics (Derio, Spain). The metabolites were extracted from 15 mg frozen liver samples by precipitating out the proteins then fractionating into pools of species with similar physicochemical properties, using appropriate combinations of water and the organic solvents methanol and chloroform as described previously^52^. The resulting mixture was homogenized using a Precellys 24 homogenizer at 6500 rpm for 45 seconds x 1 round. Samples were incubated at −20 °C for 1 hour and after vortexing them, 500 μL was collected for analysis on four platforms. Extracts were subjected to mass spectrometry coupled with ultra-high performance liquid chromatography–based analytical platforms as described in detail^53,54^. Metabolomics data were pre-processed using the TargetLynx application manager for MassLynx 4.1 (Waters Corp., Milford, MA).

### Analysis of metabolomic data

Data pre-processing generated a list of chromatographic peak areas for the metabolites detected in each sample injection. An approximated linear detection range was defined for each identified metabolite, assuming similar detector response levels for all metabolites belonging to a given chemical class represented by a single standard compound. Data points lying outside their corresponding linear detection range were replaced with missing values, and those metabolites for which more than 30% of data points were found outside their corresponding linear detection range were not used for statistical analyses. Data normalization was performed following the procedure described by Martínez-Arranz *et al*.^55^.

### Statistical analysis

For histological, routine laboratory, ELISA, PCR and protein expression analyses, data from individual mice from each group were collated and descriptive statistics generated. Data are presented as the means ± SEM. Statistical significance was analyzed using 2-tailed unpaired Student’s t test for two groups or ANOVA for multi-group analysis as appropriate for normally distributed data. Kruskal-Wallis ANOVA with multiple comparisons was performed for other numerical data what were not normally distributed. For metabolomic data, Univariate statistical analyses were also performed calculating group percentage changes and unpaired Student’s t-test p-value (or Welch’s t test where unequal variances were found). GraphPad Prism software (version 6) was used for all statistical analysis and p values < 0.05 were considered significant.

## Supplementary information

Supplementary information.
