# Supplementary material for: The PPAR α/γ Agonist Saroglitazar Improves Insulin Resistance and Steatohepatitis in a Diet Induced Animal Model of Nonalcoholic Fatty Liver Disease
Source: Sci Rep. 2020 Jun 9;10:9330. doi: 10.1038/s41598-020-66458-z (PMC7283326; doi:10.1038/s41598-020-66458-z)
Supplement: Supplementary file 1 — Supplementary information. [file 41598_2020_66458_MOESM1_ESM.pdf]

The PPAR α/γ Agonist Saroglitazar Improves Insulin Resistance and Steatohepatitis in a Diet Induced Animal Model of Nonalcoholic Fatty Liver Disease

Divya P. Kumar[1*#], **Rebecca Caffrey**[2*], Jonathon Marioneaux[2], Prasanna K. Santhekadur[1], Madhavi Bhat[2], Cristina Alonso[3], Srinivas V. Koduru[4], Binu Philip[5], Mukul R. Jain[5] , Suresh R. Giri[5], Pierre Bedossa[6] and **Arun J. Sanyal**[7#]

SUPPLEMENTARY METHODS

Blood collection and biochemical analysis. The mice were fasted overnight and the blood was collected via cardiac puncture following euthanasia. It was then centrifuged at 1500 g for 15 min at 4°C. The serum was collected and stored at -80°C for the biochemical analysis. Serum triglycerides (TG), total cholesterol (TC), non-high density lipoprotein (HDL) levels were measured using Alere Cholestech LDX analyzer, USA. Aspartate aminotransferase (AST) and alanine aminotransferase (ALT) levels were measured using Abaxis Vetscan VS2, USA following manufacturer's instructions.

Liver histology analysis. Liver histology was assessed using standard hematoxylin and eosin (H&E) stains in paraffin embedded sections. Hepatic fibrosis was assessed by picrosirius red staining and the presence of steatosis was further confirmed by Oil red O staining using established methods [1]. The liver histology was evaluated in a masked manner by an expert liver pathologist (PB) who was masked to the dietary conditions and treatment. Liver specimens were scored for the presence and severity of steatosis, hepatocellular ballooning, and inflammation as per the fatty liver inhibition of progression (FLIP) algorithm [2] and NASH-Clinical Research Network (CRN) criteria [3]. Scoring for the amount of steatosis, hepatocyte ballooning, lobular inflammation, NAFLD activity score (NAS) and fibrosis stage were carried out as described previously (21). We have previously established that the liver pathology in these mice was similar to that in humans allowing the use of these measures. The mean NAFLD activity score at the end of experiment was expected to be 5 and 80% of mice were expected to have steatohepatitis based on previous publications [25]. Based on previously reported standard deviation in scores for NAS of 10-20%, a sample size of 5-9 would be needed to show at 25-50% decrease in mean NAS with a power of 80% and p value set to 0.05. A total sample size of about 12 in each group was used. Data for vehicle control and WDSW alone were combined after they were confirmed to be similar to serve as the control for saroglitazar efficacy assessment. The study was also powered to 80% to demonstrate a decrease in proportion with steatohepatitis to 20% from 80%.

Quantitative Real-Time PCR. Total RNA was isolated from mouse liver tissue using homogenization and Direct-Zol (Zymo Research, Irvine, CA). RNA quality and concentrations were determined using the Bioanalyzer 2100 (Agilent Technologies, Santa Clara, CA). First strand cDNA was synthesized using High Capacity cDNA Reverse

Transcription kit with RNase Inhibitors (ThermoFisher, Waltham, MA) following the manufacturer's instructions. Optical density values of extracted RNA were measured using Nano Drop (ThermoFisher, Waltham, MA) to confirm an A260:A280 ratio above 1.9 and RNA integration number (RIN) was measured using BioAnalyzer (Agilent Technologies) RNA 6000 Nano Kit (RIN >8.0).

Quantitative RT-PCR was performed using an ABI QuantStudio 12KFlex Sequence Detection System (ThermoFisher, Waltham, MA). Assays were prepared using 50 ng cDNA reaction product, 2X TaqMan Gene Expression Master Mix and Assay-on-Demand primer probes (900nM unlabeled PCR primers; 250nM FAM dye-labeled TaqMan MGB probe) (ThermoFisher, Waltham, MA) in a final reaction volume of 10 µl) accordingly to the manufacturer's protocol. All the reactions were performed in triplicates. Quantitative RT-PCR conditions were 2 minutes at 50°C, 10 minutes at 95°C and 40 cycles of 15 seconds at 95°C and 1 minute at 60°C. Cycle threshold (Ct) values were obtained and the relative fold change in gene expression was calculated as 2-ΔΔCt. The change in mRNA expression was calculated using differences of Ct values compared to GAPDH (Mm99999915_g1) as endogenous control using ABI SDS 2.2.2 software [4]. The sequences of the primers used are as follows: Grp78 (Taqman Gene Expression Assays, Applied Biosystems, Mm00517691_m1); Nrf2 (Mm00477784_m1); SOD1 (Mm01344233_g1); TNFα (Mm00443258_m1); IL1-β (Mm00434228_m1); Col6a3 (Mm00711678_m1); αSMA (Mm00725412_s1); ACOX1 (Mm01246834_m1); CPT1A (Mm01231183_m1); LPIN1 (Mm00550511_m1); UCP2 (Mm00627599_m1); CD36 (Mm00432403_m1); Calnexin (Mm00500330_m1) and β-actin (Mm02619580_g1).

Western blots. Liver tissue was solubilized in RIPA buffer containing protease/phosphatase inhibitor cocktail (Cell Signaling Technology, Danvers, MA). The lysates were centrifuged at 10,000×g for 15 min at 4°C and the supernatant collected. Protein concentration was measured by BCA assay kit (Sigma Aldrich, St. Louis, MO). Equal amounts of protein were resolved by SDS-PAGE and transferred onto nitrocellulose membrane. Blots were blocked in 5% nonfat dry milk for 1 h followed by immunobloting with specific primary antibodies [anti JNK (concentration 1:1000 and Cat # 9255S, Cell Signaling); anti Erk1/2 (concentration 1:1000 and cat # 4370S, Cell Signaling); anti αSMA (concentration 1:1000 and Cat# 19245S, Cell Signaling); anti GLI1 (concentration 1:500 and Cat # sc-515781, Santa Cruz) and β-actin (concentration 1:5000 and Cat# A5441, Sigma Aldrich) at 4°C overnight and with secondary antibodies for 2 hours at room temperature. Antigen-antibody complexes were detected using Pierce ECL western blotting substrate (Thermo Scientific, Waltham, MA). Western blot images were analyzed with ImageJ software for densitometric measurements. The average intensity for each band was normalized to its respective band of β-actin.

Enzyme-linked Immunosorbent Assay (ELISA). Concentrations of Insulin (Mercodia, Uppsala, Sweden), Adiponectin (R&D systems, Minneapolis, MN) and TNF-α (R&D systems, Minneapolis, MN) were measured in the serum samples of treated and untreated mice by using ELISA kits according to manufacturer's protocol.

SUPPLEMENTARY REFERENCES

1. Mehlem, A., Hagberg, C.E., Muhl, L., Eriksson, U. & Falkevall, A. Imaging of neutral lipids by oil red O for analyzing the metabolic status in health and disease. *Nat. Protoc.* **8**, 11491154 (2013).
2. Bedossa, P. FLIP Pathology Consortium. Utility and appropriateness of the fatty liver inhibition of progression (FLIP) algorithm and steatosis, activity, and fibrosis (SAF) score in the evaluation of biopsies of non alcoholic fatty liver disease. *Hepatology*. 60, 565–575 (2014).
3. Kleiner, D.E. *et al*. Design and validation of a histological scoring system for nonalcoholic fatty liver disease. *Hepatology*. **41**, 313–1321 (2005).
4. Livak, K.J. & Schmittgen, T.D. Analysis of relative gene expression data using real time quantitative PCR and the 2 (-delta delta C(T)) method. *Methods.* **25**, 402–408 (2001).

SUPPLEMENTARY FIGURE LEGENDS

Supplementary Figure 1. Study design to evaluate the efficacy of saroglitazar in DIAMOND mice

DIAMOND mice (B6/129 mice) were fed chow diet (CDNW) or high fructose/glucose, high fat western diet (WDSW) for up to 12 weeks to develop fatty liver and steatohepatitis. Mice were administered pioglitazone, saroglitazar or vehicle control along with CDNW or WDSW for an additional 12 weeks. At the end of 24 weeks following initiation of dietary intervention and treatment, mice were fasted overnight, weighed and euthanized for blood collection and tissue harvesting.

Supplementary Figure 2. Effect of saroglitazar treatment on serum levels of adiponectin and TNFα in DIAMOND mice

DIAMOND mice fed on CDNW or WDSW were treated with saroglitazar for 12 weeks. Following treatment, adiponectin and TNFα levels were measured in the serum of DIAMOND mice. Data are expressed as the mean ± SEM for 6-12 mice per group. $^{\#}p<0.05$ compared to CDNW; $^{**}p<0.001$ compared to WDSW, vehicle control. TNFα, tumor necrosis factor alpha.

Supplementary Figure 3. Saroglitazar reduces oxidative stress and inflammatory markers in DIAMOND mice

The relative expression of hepatic mRNA levels of SOD1, TNF-α and IL1-β were measured using qRT-PCR. The experiments were carried out in triplicates and β-actin was used as endogenous control for normalizing the mRNA levels. Data are expressed as the mean ± SEM for 6-12 mice per group. $^{\#}p<0.05$, $^{\#\#}p<0.001$ compared to CDNW; $^{**}p<0.001$ compared to WDSW, vehicle control. SOD1, superoxide dismutase1; TNFα, tumor necrosis factor alpha; IL1-β, interleukin 1-beta.

Supplementary Figure 4. Saroglitazar reduces hepatic fibrosis markers in DIAMOND mice

The mRNA expression levels of col6a3 and αSMA (A and B) were measured in the liver tissue of DIAMOND mice treated with saroglitazar fed on CDNW or WDSW by qRT-PCR. The experiments were carried out in triplicates and β-actin was used as endogenous control for normalizing the mRNA levels. Morphometric analysis of collagen proportionate area (CPA) of DIAMOND mice treated with vehicle control, pioglitazone or saroglitazar for 12 weeks along with CDNW or WDSW. Data are expressed as the mean ± SEM for 6-12 mice per group. $^{\#}p<0.05$ compared to CDNW; $^{*}p<0.05$ compared to WDSW, vehicle control. col6a3, collagen type VI alpha 3 chain; αSMA, alpha smooth muscle actin.

Supplementary Figure 5. Activation of PPARα and PPARγ target genes by saroglitazar

DIAMOND mice fed on CDNW or WDSW were treated with saroglitazar for 12 weeks. Hepatic mRNA levels of PPARα target genes (ACOX1, CPT1A and LPIN1) and PPARγ target genes (UCP2 and CD36) were measured by qRT-PCR. The experiments were carried out in triplicates and Calnexin was used as endogenous control for normalizing the mRNA levels. Data are expressed as the mean ± SEM for 6-12 mice per group. $^{*}p<0.05$, $^{**}p<0.001$ compared to WDSW, vehicle control. ACOX1, acyl coenzyme A oxidase 1; CPT1A, carnitine palmitoyltransferase 1A; UCP2, uncoupling protein 2; CD36, cluster of differentiation 36.

Supplementary Figure 6. Top maps (sorted by statistically significant maps) in mice treated/untreated with saroglitazar fed on WDSW

The top (A) scored map (map with the lowest p-value) and second (B) scored map (map with the second lowest p-value) based on the enrichment distribution sorted by 'Statistically significant Maps' set. Experimental data from all files is linked to and visualized on the maps as thermometer-like figures. Up-ward thermometers have red color and indicate up-regulated signals and down-ward (blue) ones indicate down-regulated expression levels of the genes. Analyzed using MetaCore pathway analysis- version 6.5 (Clarivate Analytics, New York, NY- https://portal.genego.com).

Supplementary Figure 7. Top scored networks in mice treated/untreated with saroglitazar fed on WDSW

The top (A) scored (by the number of pathways) and second scored (by the number of pathways) AN network in mice treated/untreated with saroglitazar fed on WDSW gene list. Thick cyan lines indicate the fragments of canonical pathways. Up-regulated genes are marked with red circles; down-regulated with blue circles. The 'checkerboard' color indicates mixed expression for the gene between files or between multiple tags for the same gene. Biological pathway interactions of mRNA expressions were analyzed using MetaCore pathway analysis (version 6.5) of differentially expressed genes (Clarivate Analytics, New York, NY- https://portal.genego.com) with $p<0.05$ and greater than two-fold change.

Supplementary Figure 8. Effect of saroglitazar on the liver metabolome of DIAMOND mice

Mass spectrometry coupled to ultra-high performance liquid chromatography (UHPLC-MS) was used to determine the metabolites from the liver samples of mice treated/untreated with saroglitazar fed on CDNW or WDSW. Box plots of (A) diglycerides (32:0), (B) diglycerides (36:1), (C) amino acids, (D) nucleotides and nucleosides. Data are expressed as the mean ± SEM for 6-12 mice per group. $^{\#\#}p<0.001$ compared to CDNW; $^{*}p<0.05$, $^{**}p<0.001$ compared to WDSW, vehicle control.

Supplementary Figure 9. Original scans of western blot images presented in the Figure 5C-5F. The molecular weight of the detected proteins are pJNK- 54/46 kDa, pERK1/2- 44/42 kDa, αSMA- 42 kDa, GLI1- 118 kDa and β-actin- 42 kDa.

Supplementary Table 1. Differential gene expression in saroglitazar treated vs. untreated mice

Mice fed on WDSW were either treated or untreated with saroglitazar. Differentially expressed hepatic mRNA were quantified (FDR<0.05) and the genes upregulated or down regulated listed.

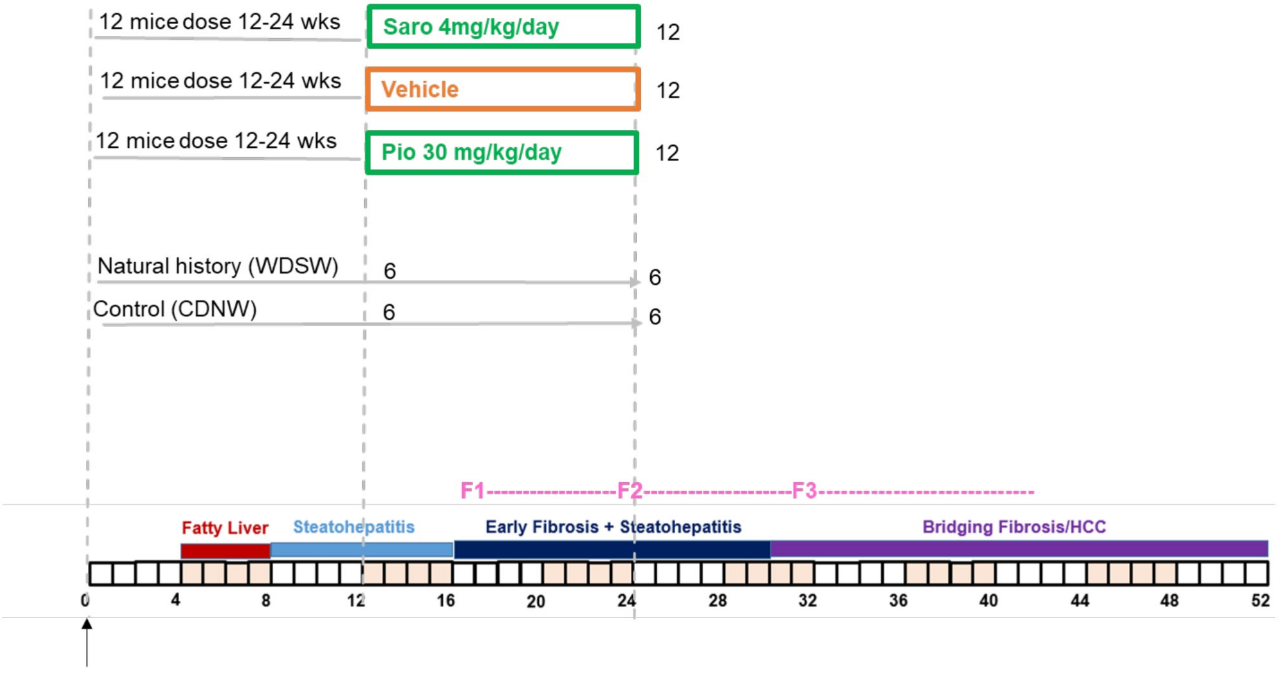

Begin DIAMOND diet
Mice are 8-12 weeks prior to starting the diet

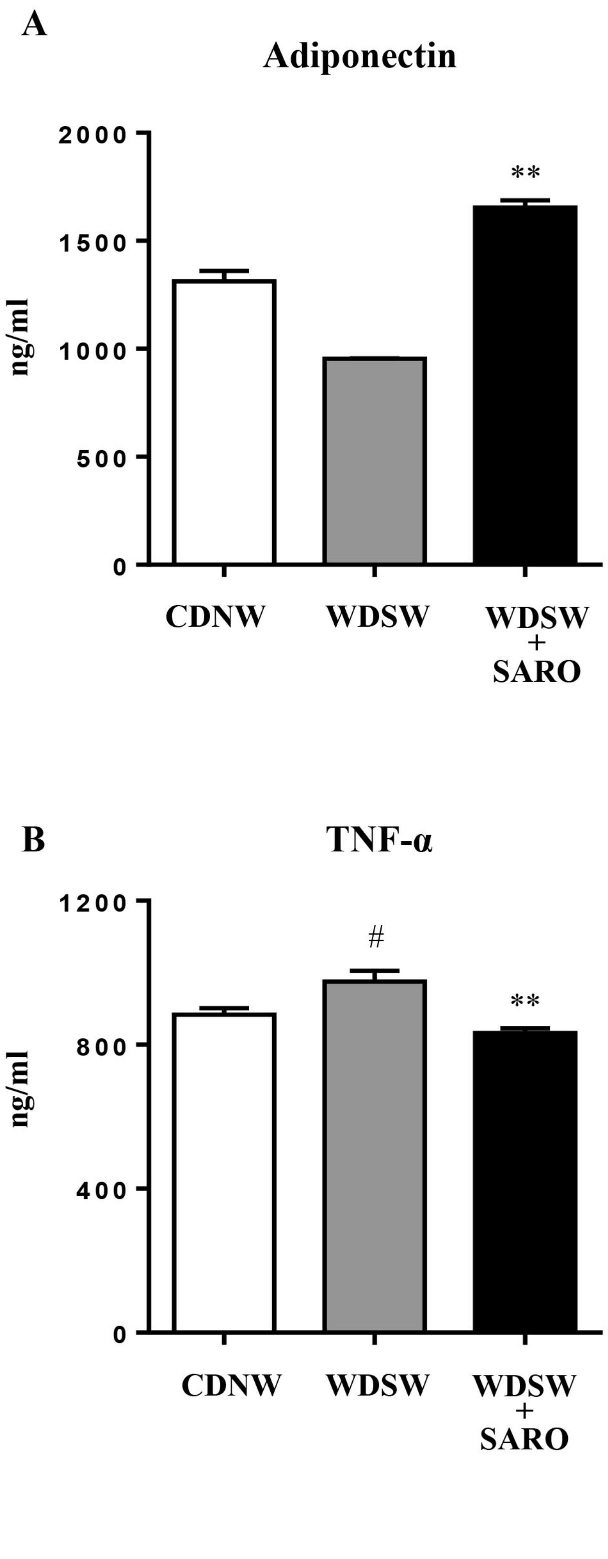
A
Adiponectin
ng/ml
0
500
1000
1500
2000
**
CDNW
WDSW
WDSW
+
SARO
B
TNF-α
ng/ml
0
400
800
1200
#
**
CDNW
WDSW
WDSW
+
SARO

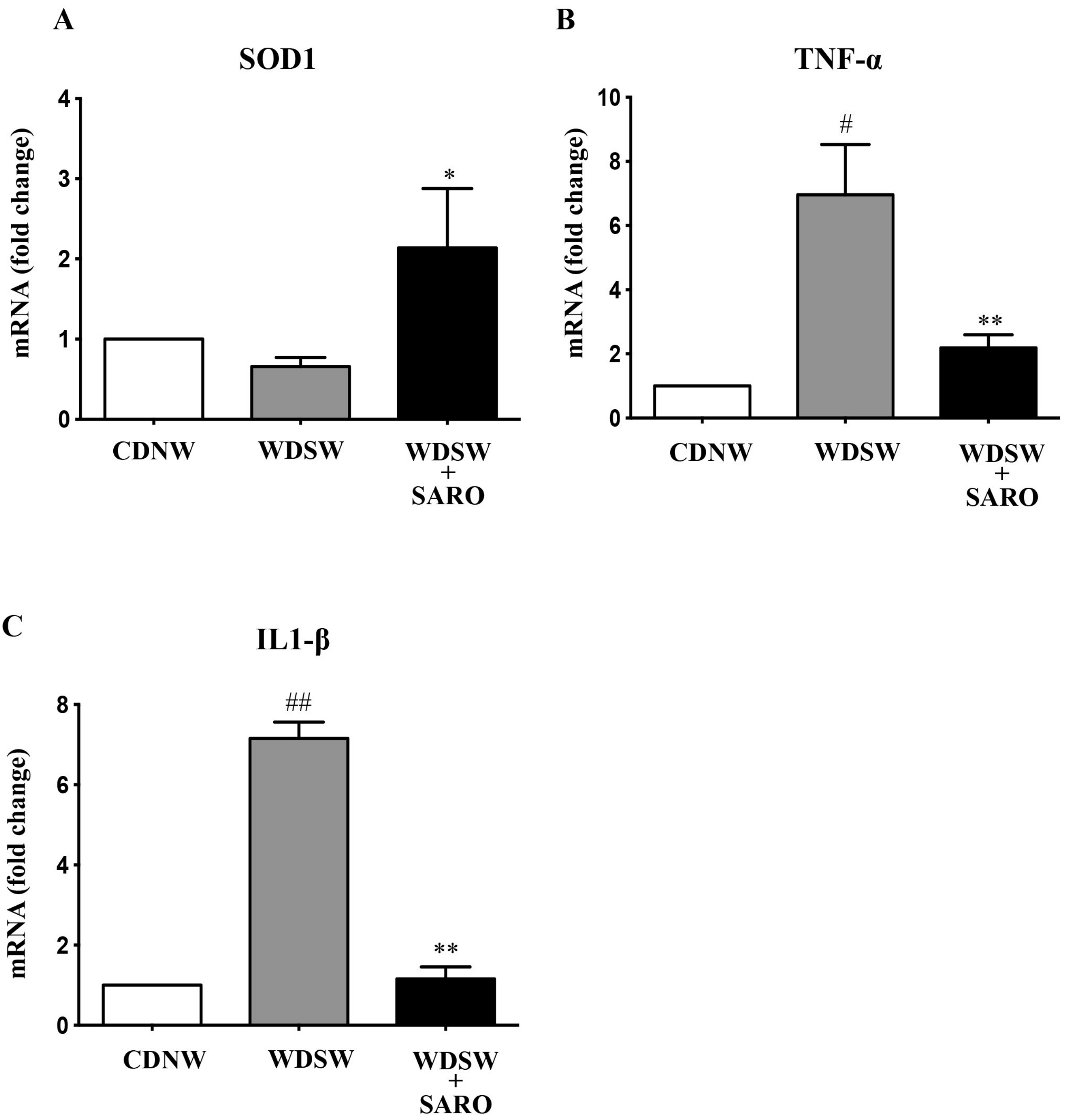
A
SOD1
mRNA (fold change)
0
1
2
3
4
*
CDNW
WDSW
WDSW
+
SARO
B
TNF-α
mRNA (fold change)
0
2
4
6
8
10
#
**
CDNW
WDSW
WDSW
+
SARO
C
IL1-β
mRNA (fold change)
0
2
4
6
8
##
**
CDNW
WDSW
WDSW
+
SARO

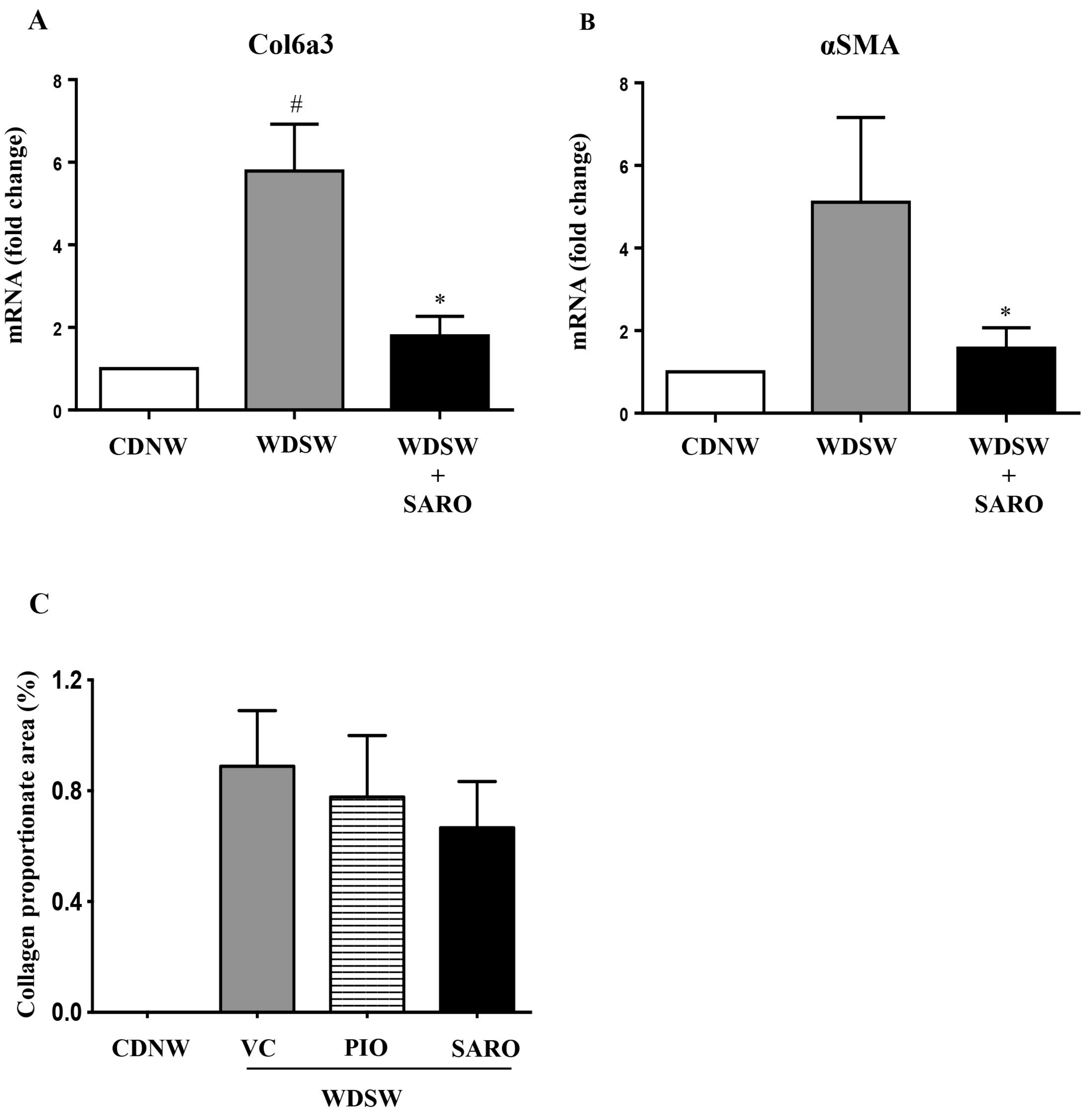
A
Col6a3
mRNA (fold change)
0
2
4
6
8
#
*
CDNW
WDSW
WDSW
+
SARO
B
αSMA
mRNA (fold change)
0
2
4
6
8
*
CDNW
WDSW
WDSW
+
SARO
C
Collagen proportionate area (%)
0.0
0.4
0.8
1.2
CDNW
VC
PIO
SARO
WDSW

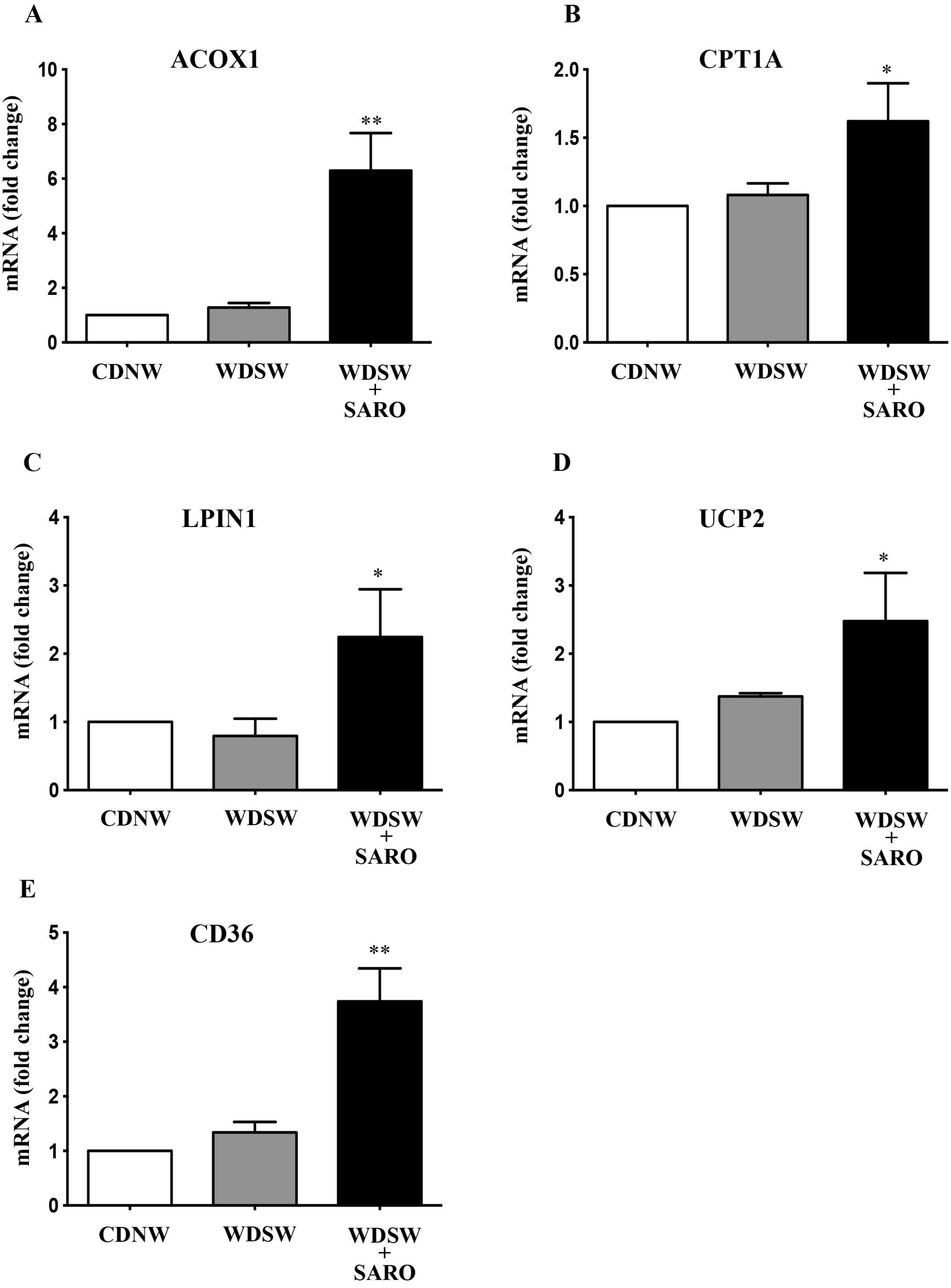

A
ACOX1
B
CPT1A
C
LPIN1
D
UCP2
E
CD36
mRNA (fold change)
**
*
CDNW
WDSW
WDSW + SARO

A

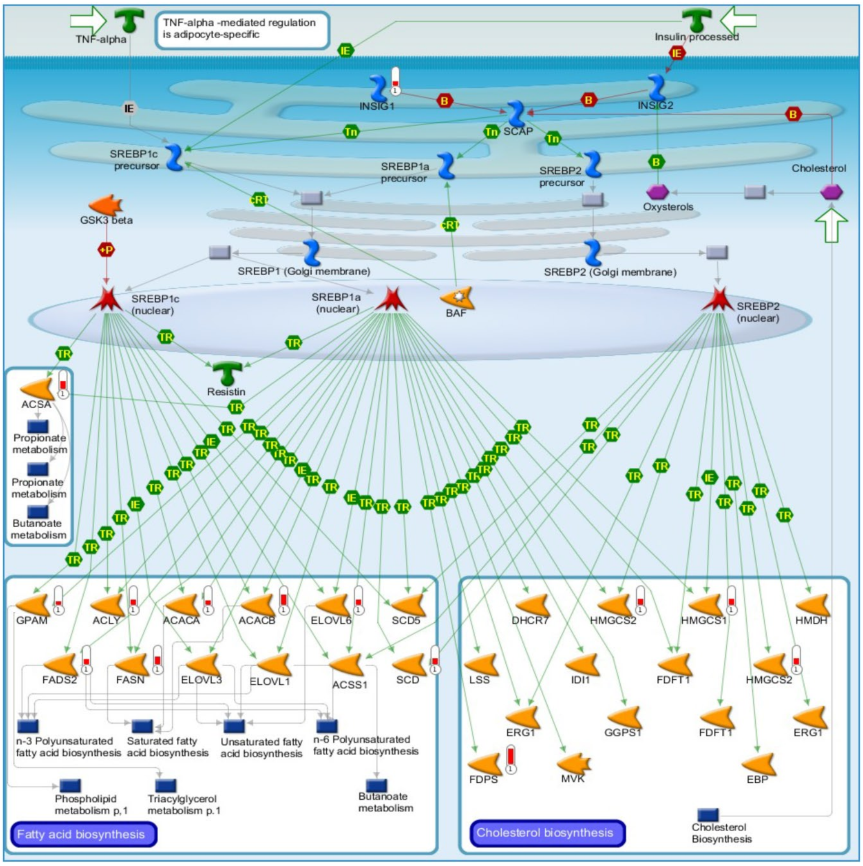

SCAP/SREBP Transcriptional Control of Cholesterol and FA Biosynthesis

B

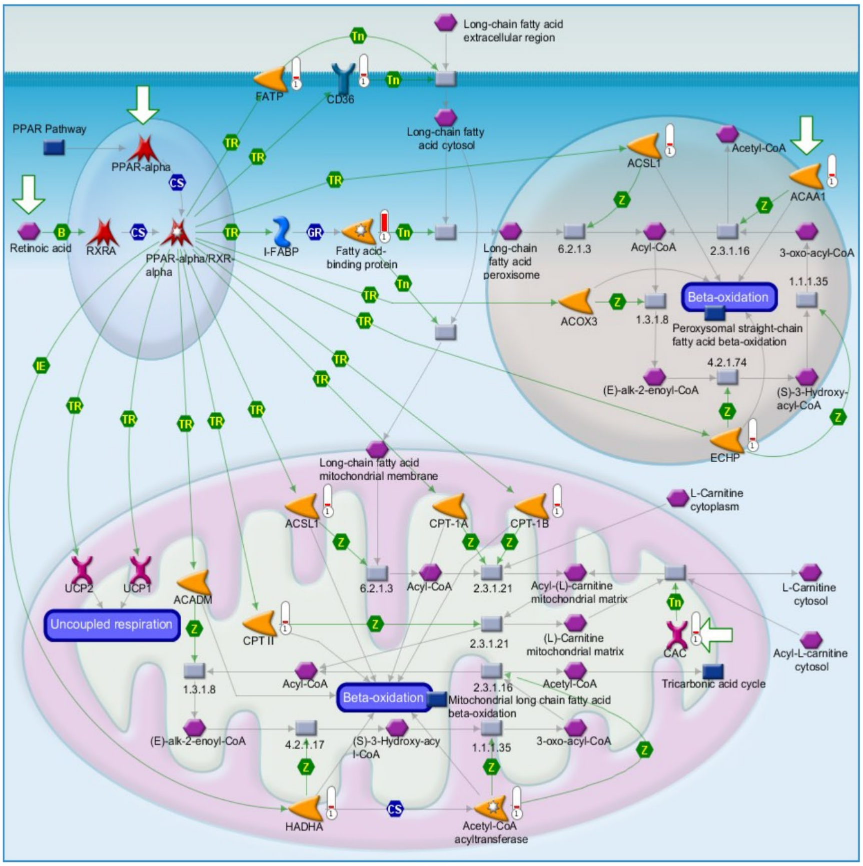

Regulation of Lipid Metabolism_PPAR Regulation of Lipid Metabolism

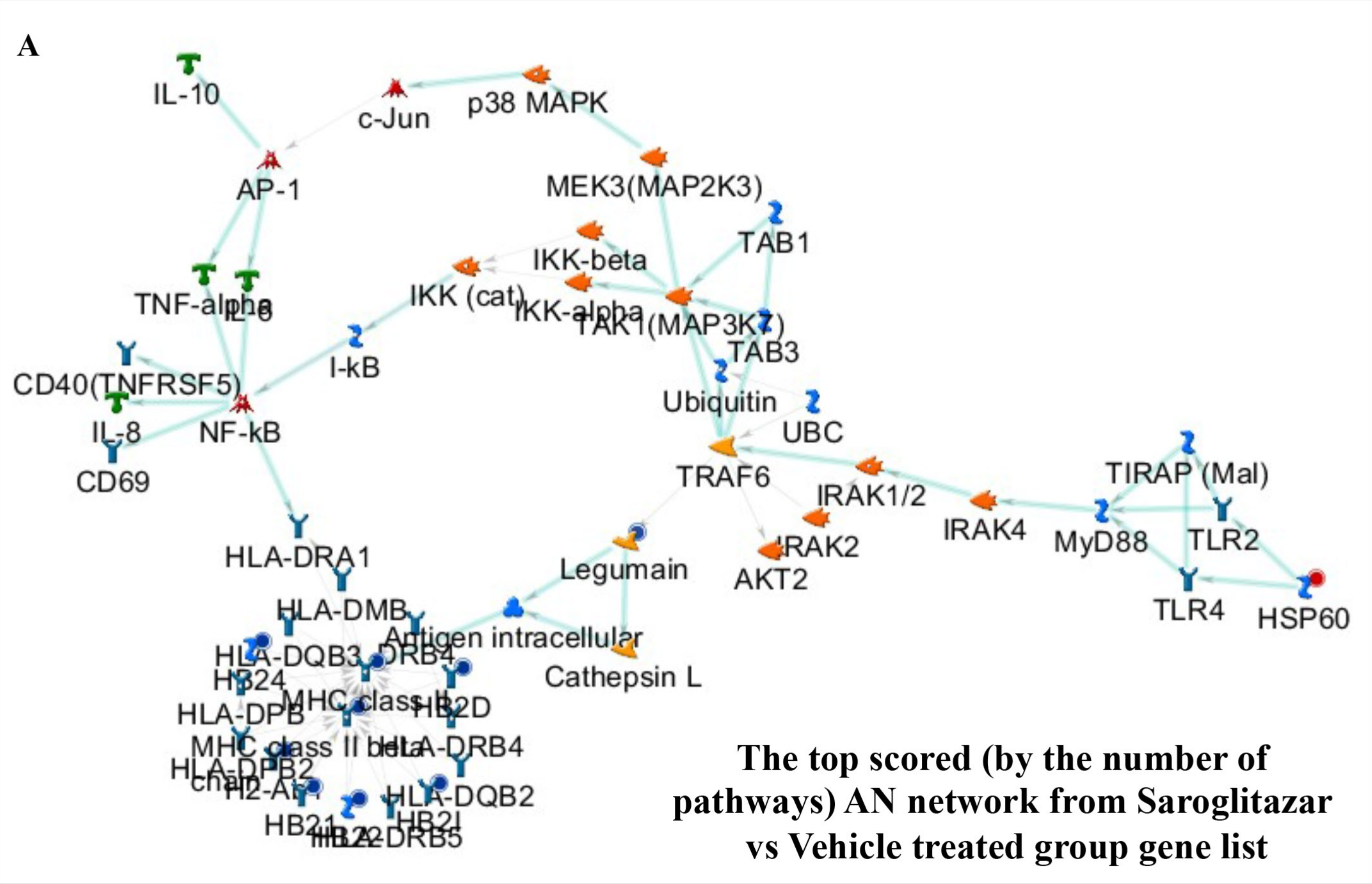

The top scored (by the number of pathways) AN network from Saroglitazar vs Vehicle treated group gene list

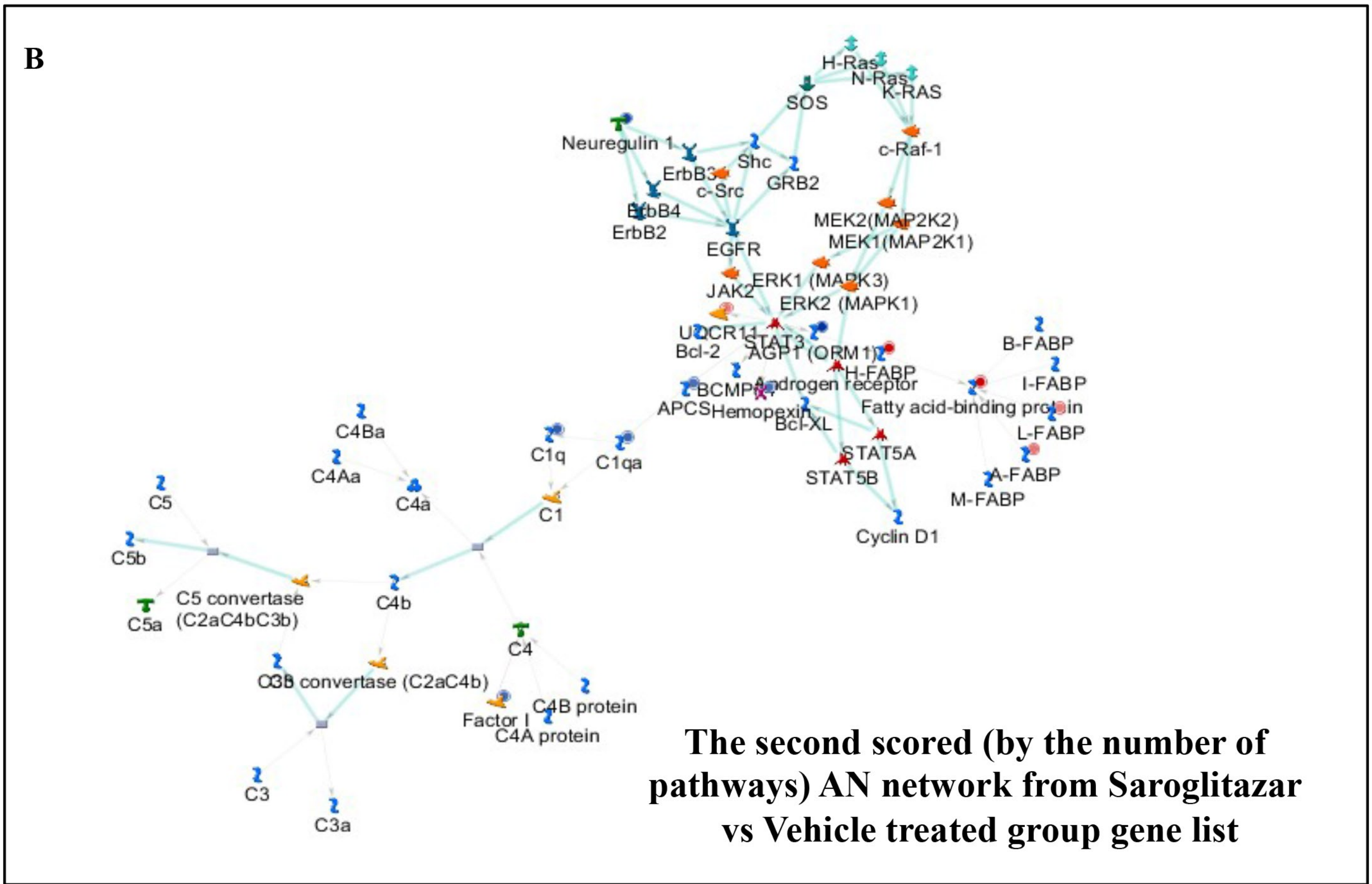

The second scored (by the number of pathways) AN network from Saroglitazar vs Vehicle treated group gene list

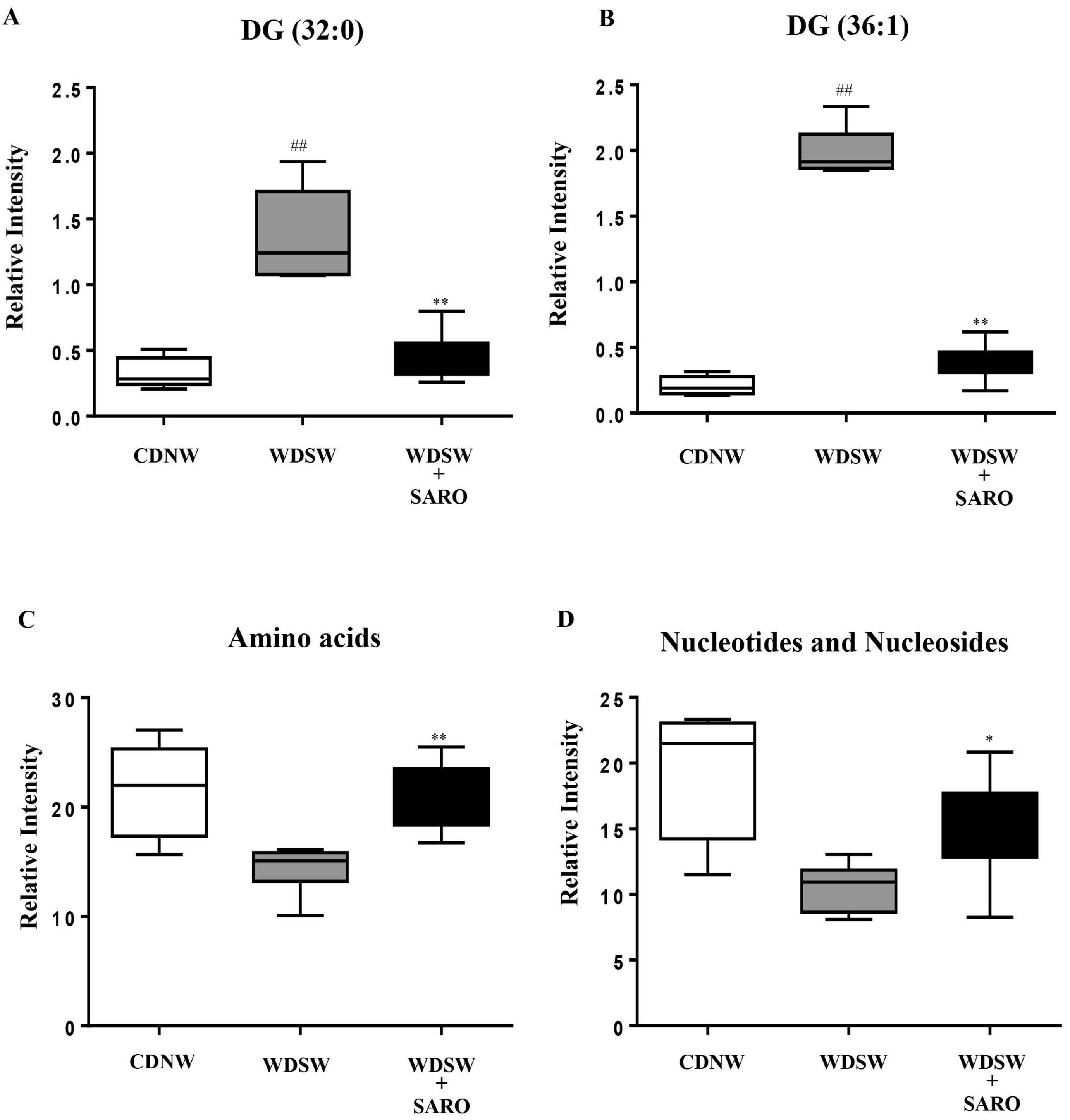
A
DG (32:0)
Relative Intensity
2.5
2.0
1.5
1.0
0.5
0.0
##
**
CDNW
WDSW
WDSW
+
SARO
B
DG (36:1)
Relative Intensity
2.5
2.0
1.5
1.0
0.5
0.0
##
**
CDNW
WDSW
WDSW
+
SARO
C
Amino acids
Relative Intensity
30
20
10
0
**
CDNW
WDSW
WDSW
+
SARO
D
Nucleotides and Nucleosides
Relative Intensity
25
20
15
10
5
0
*
CDNW
WDSW
WDSW
+
SARO

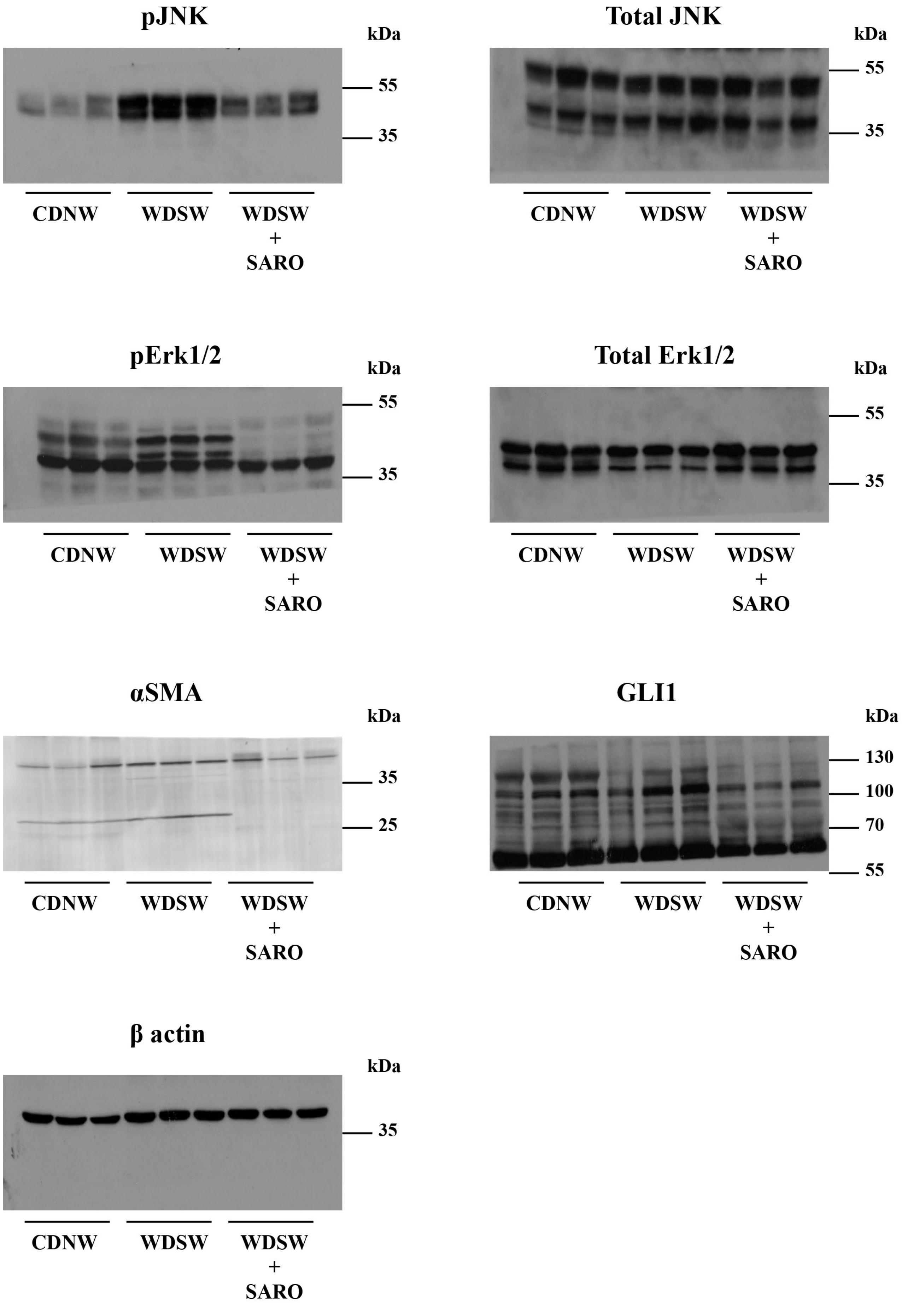
pJNK
kDa
55
35
CDNW
WDSW
WDSW + SARO
Total JNK
kDa
55
35
CDNW
WDSW
WDSW + SARO
pErk1/2
kDa
55
35
CDNW
WDSW
WDSW + SARO
Total Erk1/2
kDa
55
35
CDNW
WDSW
WDSW + SARO
αSMA
kDa
35
25
CDNW
WDSW
WDSW + SARO
GLI1
kDa
130
100
70
55
CDNW
WDSW
WDSW + SARO
β actin
kDa
35
CDNW
WDSW
WDSW + SARO

Supplementary Table 1: Saroglitazar vs WDSW gene list

Gene ID	Fold Change	FDR	P-value
Fabp3	537.76	8.24E-14	9.99E-17
Gm15441	140.80	6.65E-15	4.60E-18
Cyp4a31	70.76	1.24E-15	2.15E-19
Acot1	51.93	6.65E-15	4.57E-18
Cyp4a14	51.26	6.31E-15	3.28E-18
Cpt1b	39.19	8.56E-12	1.70E-14
Fabp4	36.02	1.88E-14	1.79E-17
Ehhadh	33.49	3.80E-15	1.31E-18
Fabp5	23.68	2.49E-14	2.80E-17
Cidea	23.58	6.55E-10	2.92E-12
Acot2	17.93	1.30E-13	1.69E-16
Cyp4a12b	17.57	2.85E-11	6.40E-14
Cyp4a10	17.49	3.66E-13	5.06E-16
Me1	16.69	6.31E-15	3.22E-18
Pdk4	12.77	1.27E-08	1.06E-10
Fdps	12.69	6.90E-11	2.03E-13
Spon2	12.18	2.04E-10	6.52E-13
Slc27a1	12.11	5.57E-11	1.49E-13
Pla2g7	11.53	2.64E-11	5.70E-14
Cyp2a4	9.25	6.55E-10	3.06E-12
Acacb	8.23	2.75E-09	1.67E-11
Acot3	7.63	3.09E-07	4.26E-09
Scd2	7.39	1.06E-08	8.35E-11
Dbi	7.29	3.44E-09	2.20E-11
Serinc2	6.91	1.50E-08	1.33E-10
Cyp2b9	6.49	3.51E-09	2.27E-11
Fabp1	6.37	3.08E-09	1.89E-11
Hsd17b11	6.35	1.36E-09	7.31E-12
Acss2	6.18	4.65E-10	1.88E-12
Fasn	6.00	1.00E-08	7.81E-11
Raet1d	5.47	3.21E-06	7.07E-08
Cyp4a32	5.46	1.29E-08	1.08E-10
Retsat	5.36	1.53E-09	8.48E-12
Acsl5	5.32	8.86E-10	4.44E-12
Fads1	5.24	2.74E-08	2.63E-10
Acox1	5.04	4.66E-09	3.30E-11
Scd1	4.87	3.95E-06	9.16E-08
Igfbp2	4.64	2.51E-08	2.37E-10

Hmgcs1	4.59	4.60E-09	3.22E-11
Slc16a1	4.50	3.69E-08	3.70E-10
Fads2	4.29	4.35E-08	4.55E-10
Elovl6	4.27	2.62E-07	3.52E-09
Pltp	4.05	1.20E-06	2.16E-08
Crat	4.04	4.51E-07	6.75E-09
Abcd3	3.92	4.39E-08	4.63E-10
Aspg	3.91	3.09E-07	4.27E-09
Acly	3.89	4.49E-07	6.69E-09
Ctse	3.86	6.58E-08	7.17E-10
Gstm4	3.82	6.77E-08	7.44E-10
Csad	3.76	7.89E-07	1.38E-08
Vnn1	3.75	8.37E-08	9.70E-10
Gstt2	3.71	1.73E-07	2.18E-09
Hacl1	3.65	9.21E-06	2.47E-07
Fam213a	3.65	2.05E-07	2.63E-09
Elovl5	3.54	4.36E-07	6.44E-09
Hadhb	3.45	1.28E-07	1.56E-09
Gm4952	3.43	6.37E-07	1.01E-08
Acaca	3.35	7.29E-07	1.24E-08
Ly6d	3.35	6.40E-04	4.42E-05
Acaa1b	3.30	1.89E-06	3.76E-08
Paqr9	3.22	4.87E-07	7.33E-09
Rdh16	3.16	8.40E-08	9.81E-10
Lpl	3.08	1.34E-05	3.96E-07
Hsdl2	3.03	2.34E-06	4.86E-08
Decr1	3.03	3.58E-07	5.08E-09
Plin2	3.02	1.27E-05	3.69E-07
Insig1	3.01	2.87E-06	6.15E-08
Eci1	2.99	3.90E-07	5.63E-09
Mgll	2.99	1.89E-06	3.74E-08
Hadha	2.98	1.84E-06	3.60E-08
Acat2	2.95	5.77E-07	9.03E-09
Sc5d	2.89	5.35E-07	8.15E-09
Eci2	2.87	2.29E-06	4.74E-08
Fbxo21	2.83	1.10E-06	1.97E-08
Pctp	2.83	1.22E-06	2.23E-08
Grhpr	2.83	5.24E-06	1.27E-07
Sigmar1	2.72	5.76E-06	1.42E-07
Pnpla2	2.71	1.17E-05	3.38E-07
Acsl1	2.71	1.61E-05	5.01E-07

Crot	2.71	8.97E-05	4.13E-06
Gpam	2.70	4.63E-06	1.11E-07
Acadl	2.67	2.13E-06	4.38E-08
Ech1	2.67	2.82E-06	5.97E-08
Acsl4	2.62	6.44E-05	2.75E-06
Lpcat3	2.58	9.21E-06	2.48E-07
Cd36	2.56	4.40E-05	1.73E-06
Mttp	2.55	1.07E-05	3.00E-07
Cops6	2.51	7.38E-06	1.89E-07
Adtrp	2.49	4.78E-06	1.15E-07
Pex11a	2.45	1.26E-04	6.17E-06
Nr1d1	2.42	1.58E-03	1.33E-04
Ephx2	2.40	1.47E-05	4.38E-07
Cbr1	2.38	7.09E-05	3.07E-06
Slc25a20	2.38	1.42E-05	4.21E-07
Vwa8	2.37	8.08E-05	3.64E-06
Chchd10	2.36	2.44E-04	1.39E-05
Cycs	2.35	1.09E-05	3.05E-07
Acadvl	2.35	2.56E-05	8.69E-07
Oplah	2.31	1.64E-04	8.62E-06
Mif	2.31	3.95E-04	2.42E-05
Pgd	2.29	5.02E-04	3.25E-05
Etfdh	2.27	2.75E-05	9.48E-07
Psmd4	2.26	1.19E-04	5.82E-06
Mtch2	2.24	1.67E-05	5.30E-07
Pank1	2.23	3.21E-04	1.91E-05
H2-Q10	2.22	1.26E-04	6.19E-06
Hspd1	2.21	3.52E-05	1.32E-06
Slc3a2	2.20	1.09E-03	8.44E-05
Tecr	2.20	9.78E-05	4.60E-06
Ddt	2.18	1.41E-04	7.11E-06
Cyp8b1	2.18	2.37E-03	2.21E-04
Elovl2	2.17	4.89E-05	1.97E-06
Uqcr11	2.16	6.44E-05	2.76E-06
Lgals9	2.15	1.04E-04	4.96E-06
Aldh3a2	2.13	8.95E-04	6.60E-05
Chchd2	2.11	6.17E-05	2.59E-06
Cpt2	2.10	1.19E-04	5.83E-06
St3gal5	2.09	5.20E-04	3.40E-05
Cisd1	2.09	2.36E-04	1.33E-05
Rplp2-ps1	2.07	6.36E-03	7.53E-04

Ran	2.06	2.42E-04	1.38E-05
Rps26	2.06	7.17E-04	5.05E-05
Chchd3	2.06	1.39E-04	6.93E-06
Cs	2.05	1.42E-04	7.19E-06
G0s2	2.04	4.68E-03	5.12E-04
Sdhd	2.03	8.16E-05	3.71E-06
Cpn1	2.03	3.21E-04	1.91E-05
Cox8a	2.03	1.95E-04	1.05E-05
Atp5g3	2.03	1.56E-04	8.12E-06
Ndufb10	2.03	4.64E-04	2.95E-05
Pnkd	2.03	3.97E-04	2.43E-05
Mlycd	2.02	1.36E-04	6.78E-06
Cox6b1	2.01	2.17E-04	1.20E-05
Acsf2	2.00	4.43E-04	2.79E-05
Qdpr	1.98	7.23E-04	5.12E-05
Rps8	1.98	3.35E-03	3.39E-04
Eef1b2	1.96	1.91E-03	1.68E-04
Hsd17b12	1.96	2.48E-04	1.42E-05
Decr2	1.96	2.16E-04	1.19E-05
Saraf	1.96	1.95E-04	1.05E-05
Cox6a1	1.95	7.67E-04	5.48E-05
BC004004	1.95	8.90E-04	6.55E-05
Hacd3	1.94	4.67E-04	2.97E-05
Pex19	1.93	2.80E-04	1.63E-05
Mmd	1.93	4.11E-04	2.56E-05
Bsg	1.93	7.57E-04	5.40E-05
Eif4a2	1.93	1.63E-03	1.38E-04
Rtf2	1.93	5.07E-03	5.70E-04
Ube2l6	1.92	3.62E-04	2.18E-05
Phb2	1.92	1.35E-03	1.09E-04
Etfb	1.91	9.03E-04	6.67E-05
Rps7	1.90	1.03E-03	7.79E-05
Snrk	1.90	1.09E-03	8.36E-05
Ndufb8	1.90	7.38E-04	5.23E-05
Hmgcs2	1.89	2.06E-03	1.86E-04
Slc27a2	1.89	1.01E-03	7.65E-05
Atp5o	1.88	7.43E-04	5.29E-05
Tmem135	1.87	7.08E-04	4.98E-05
Oaz1-ps	1.87	1.45E-02	2.10E-03
Gaa	1.86	2.49E-03	2.36E-04
Aldh9a1	1.86	3.43E-04	2.06E-05

Adipor2	1.86	1.72E-03	1.48E-04
Scap	1.86	2.27E-03	2.10E-04
Slc4a4	1.85	1.47E-02	2.14E-03
Mdh2	1.85	9.38E-04	7.00E-05
Cox7b	1.85	7.71E-04	5.52E-05
Ebp	1.85	1.37E-03	1.11E-04
Aco2	1.84	1.07E-03	8.14E-05
Tymp	1.84	6.95E-03	8.45E-04
Acaa2	1.84	1.57E-03	1.32E-04
Cox5a	1.84	2.39E-03	2.24E-04
Cmbl	1.83	1.11E-03	8.63E-05
Dhrs4	1.83	1.09E-03	8.40E-05
Phb	1.83	8.27E-04	6.04E-05
Acad11	1.83	3.56E-03	3.66E-04
Acat1	1.82	1.53E-03	1.28E-04
Atp5g1	1.82	6.16E-04	4.21E-05
Ndufb9	1.82	1.42E-03	1.17E-04
Hmgcl	1.81	3.18E-03	3.18E-04
Coq8a	1.81	3.80E-03	3.94E-04
2900026A02Rik	1.81	1.91E-03	1.68E-04
Rps14	1.81	3.38E-03	3.42E-04
Cox4i1	1.80	2.92E-03	2.88E-04
Uqcrb	1.79	2.14E-03	1.95E-04
Lonp2	1.79	2.73E-03	2.65E-04
Rps18	1.79	2.31E-03	2.14E-04
Cyb5b	1.79	6.51E-04	4.51E-05
Rpl35	1.79	3.13E-03	3.12E-04
Tgoln1	1.78	5.44E-03	6.25E-04
Cyc1	1.78	2.20E-03	2.01E-04
Rps5	1.77	5.38E-03	6.16E-04
Cox5b	1.76	2.55E-03	2.45E-04
Cox6c	1.76	2.13E-03	1.93E-04
Tmed5	1.75	1.51E-02	2.22E-03
Dlst	1.75	1.53E-03	1.27E-04
Atp5d	1.75	5.25E-03	5.97E-04
Idh1	1.74	3.51E-03	3.59E-04
Hsp90aa1	1.74	1.22E-02	1.69E-03
Cyp2j5	1.73	5.77E-03	6.67E-04
Col4a2	1.73	2.77E-02	4.76E-03
Atp5l	1.73	2.00E-03	1.80E-04
Rpl13	1.72	6.37E-03	7.55E-04

Tpt1	1.72	6.95E-03	8.45E-04
Rpl10a	1.71	8.65E-03	1.12E-03
Rps15	1.71	1.31E-02	1.86E-03
Shmt1	1.70	3.00E-03	2.97E-04
Rpl14-ps1	1.70	1.36E-02	1.94E-03
Cluh	1.69	6.05E-03	7.08E-04
Uba52	1.69	7.58E-03	9.46E-04
Atp5f1	1.69	2.82E-03	2.76E-04
Cyp4a12a	1.69	5.97E-03	6.95E-04
Acot12	1.67	6.97E-03	8.48E-04
Ndufa4	1.67	4.36E-03	4.72E-04
Neat1	1.67	1.61E-02	2.41E-03
Rpl18a	1.67	1.12E-02	1.53E-03
Rpl27	1.66	1.62E-02	2.42E-03
Rps24	1.66	1.18E-02	1.63E-03
Ndufv2	1.66	4.74E-03	5.21E-04
Rplp1	1.65	7.22E-03	8.89E-04
01-Mar	1.65	3.47E-03	3.55E-04
Ces1f	1.65	7.76E-03	9.75E-04
Tkt	1.65	8.57E-03	1.11E-03
Hspa9	1.64	7.06E-03	8.62E-04
Ces1e	1.64	7.01E-03	8.54E-04
Slc25a42	1.64	7.07E-03	8.64E-04
Fau	1.64	2.01E-02	3.17E-03
Rpl19	1.64	1.47E-02	2.14E-03
Rps27a	1.64	1.98E-02	3.12E-03
Plbd1	1.63	1.24E-02	1.74E-03
Atp5c1	1.63	4.69E-03	5.13E-04
Map1lc3b	1.63	4.02E-03	4.23E-04
Rpl23a	1.62	7.81E-03	9.82E-04
Rack1	1.62	1.27E-02	1.78E-03
Hsd17b4	1.62	9.16E-03	1.20E-03
Atp5g2	1.62	1.51E-02	2.23E-03
Psmc1	1.62	1.12E-02	1.54E-03
Slc25a22	1.62	1.13E-02	1.55E-03
Rpl18	1.62	2.01E-02	3.16E-03
Acaa1a	1.62	1.78E-02	2.73E-03
Uqcrq	1.62	5.70E-03	6.59E-04
Ghitm	1.62	9.16E-03	1.20E-03
Pcbd1	1.61	7.37E-03	9.12E-04
1110008F13Rik	1.61	7.51E-03	9.36E-04

Rpl32	1.61	1.97E-02	3.10E-03
Rpl28	1.61	2.67E-02	4.54E-03
Stt3b	1.61	1.82E-02	2.80E-03
Akap1	1.60	5.13E-03	5.79E-04
Bst2	1.60	2.40E-02	4.00E-03
Rtn4	1.60	1.21E-02	1.68E-03
Prodh2	1.60	2.33E-02	3.85E-03
Pnpla7	1.60	2.11E-02	3.38E-03
Eef1d	1.60	2.95E-02	5.14E-03
Gm15501	1.60	3.05E-02	5.38E-03
Gm15772	1.60	1.87E-02	2.90E-03
Psmb1	1.59	9.06E-03	1.18E-03
Fcgrt	1.58	2.07E-02	3.30E-03
Sar1b	1.58	1.00E-02	1.34E-03
Uqcrfs1	1.58	4.99E-03	5.55E-04
Rps16	1.58	2.01E-02	3.17E-03
Acadm	1.58	2.04E-02	3.24E-03
Eps8l2	1.56	3.88E-02	7.43E-03
Slc25a1	1.56	4.63E-02	9.29E-03
Rpl13a	1.56	2.11E-02	3.39E-03
Pgam1	1.55	1.12E-02	1.53E-03
Rps9	1.55	4.16E-02	8.11E-03
Psma4	1.55	1.06E-02	1.43E-03
Rps3	1.55	1.40E-02	2.01E-03
Cyp3a13	1.55	3.14E-02	5.59E-03
Slc25a3	1.55	1.86E-02	2.88E-03
Gstm1	1.54	4.31E-02	8.47E-03
Arg1	1.54	1.70E-02	2.57E-03
Grpel1	1.54	1.51E-02	2.22E-03
Atp5a1	1.54	1.31E-02	1.85E-03
Cox7a2l	1.54	2.35E-02	3.90E-03
Pcyox1	1.53	1.54E-02	2.28E-03
Rps10	1.53	3.38E-02	6.14E-03
Rpl27a	1.53	4.36E-02	8.61E-03
Hint1	1.53	2.13E-02	3.42E-03
Rplp0	1.52	3.25E-02	5.85E-03
Pabpc1	1.52	4.99E-02	1.02E-02
Samm50	1.52	2.17E-02	3.51E-03
Adh1	1.52	4.67E-02	9.39E-03
Creg1	1.51	1.50E-02	2.21E-03
Rpl37rt	1.51	3.51E-02	6.46E-03

Rpl7a	1.51	2.94E-02	5.13E-03
Rps17	1.51	4.43E-02	8.76E-03
Atp5b	1.51	2.16E-02	3.48E-03
Eef1g	1.51	3.93E-02	7.54E-03
St6gal1	1.50	2.19E-02	3.55E-03
Ndufs2	1.50	2.17E-02	3.52E-03
Sod1	1.49	4.27E-02	8.37E-03
Agpat3	1.49	2.96E-02	5.17E-03
Rpl23	1.49	3.29E-02	5.93E-03
Uqcrc2	1.48	2.03E-02	3.21E-03
Urah	1.48	3.37E-02	6.11E-03
Ppia	1.48	3.39E-02	6.16E-03
Rpl15	1.48	2.08E-02	3.32E-03
Ndufa10	1.48	4.58E-02	9.14E-03
Suox	1.48	2.53E-02	4.25E-03
Sdhc	1.48	3.71E-02	6.91E-03
Psmd1	1.47	4.13E-02	8.03E-03
Ccni	1.47	4.13E-02	8.03E-03
Cnbp	1.46	4.88E-02	9.92E-03
Pgk1	1.46	3.40E-02	6.20E-03
Rpl11	1.45	4.83E-02	9.78E-03
Ptp4a1	-1.40	3.89E-02	7.46E-03
1300017J02Rik	-1.40	4.05E-02	7.86E-03
Slc25a23	-1.41	4.83E-02	9.79E-03
Sephs2	-1.43	3.42E-02	6.23E-03
Itih1	-1.43	3.78E-02	7.13E-03
Vtn	-1.44	4.85E-02	9.84E-03
Ak2	-1.44	4.20E-02	8.21E-03
Dmgdh	-1.45	2.93E-02	5.10E-03
B2m	-1.46	4.31E-02	8.48E-03
Atp1a1	-1.46	4.39E-02	8.68E-03
C130074G19Rik	-1.47	4.62E-02	9.23E-03
Agt	-1.47	3.67E-02	6.81E-03
Cxcl12	-1.48	3.17E-02	5.64E-03
Cyp27a1	-1.48	2.96E-02	5.18E-03
Aldh1l1	-1.49	4.01E-02	7.77E-03
Apoc3	-1.50	2.57E-02	4.34E-03
Apon	-1.51	2.28E-02	3.73E-03
Mcfd2	-1.51	1.27E-02	1.79E-03
Cyp2d22	-1.51	1.60E-02	2.38E-03
Nudt4	-1.51	2.30E-02	3.78E-03

Ctsh	-1.52	1.66E-02	2.49E-03
Sdc4	-1.52	2.51E-02	4.22E-03
Cap1	-1.52	4.24E-02	8.31E-03
Man2a1	-1.53	3.33E-02	6.02E-03
Mcl1	-1.53	1.16E-02	1.59E-03
Apoc2	-1.53	1.89E-02	2.94E-03
Cpn2	-1.53	1.26E-02	1.77E-03
Aplp2	-1.54	4.31E-02	8.47E-03
Pcsk6	-1.54	3.91E-02	7.49E-03
Gpt	-1.55	1.92E-02	2.99E-03
Itm2b	-1.55	1.14E-02	1.56E-03
Glyctk	-1.56	1.00E-02	1.34E-03
Igfbp7	-1.57	4.63E-02	9.27E-03
Lrp1	-1.57	2.22E-02	3.62E-03
Proz	-1.58	8.61E-03	1.11E-03
Fn1	-1.58	2.28E-02	3.73E-03
Ang	-1.58	2.92E-02	5.09E-03
Tkfc	-1.59	1.63E-02	2.45E-03
Lgals8	-1.59	6.41E-03	7.63E-04
Cnn3	-1.59	7.18E-03	8.80E-04
Ahsg	-1.59	1.98E-02	3.11E-03
Azgp1	-1.60	1.10E-02	1.49E-03
Calm1	-1.61	1.66E-02	2.50E-03
Abat	-1.61	9.20E-03	1.21E-03
Gm21320	-1.61	4.11E-02	7.98E-03
Zfp36	-1.62	3.83E-02	7.30E-03
Slc22a1	-1.63	6.79E-03	8.19E-04
Qsox1	-1.63	5.39E-03	6.19E-04
Slc2a2	-1.63	2.37E-02	3.94E-03
Hs6st1	-1.63	4.63E-03	5.06E-04
Ambp	-1.65	6.15E-03	7.22E-04
Gsta3	-1.65	9.25E-03	1.22E-03
Akr1c14	-1.67	1.48E-02	2.17E-03
Herpud1	-1.67	6.34E-03	7.50E-04
Apob	-1.68	1.34E-02	1.92E-03
Aldh2	-1.68	2.90E-03	2.85E-04
Cyp3a25	-1.68	3.11E-03	3.10E-04
Gm2a	-1.69	6.69E-03	8.05E-04
Sqor	-1.69	2.50E-03	2.37E-04
Errfi1	-1.69	2.40E-02	4.00E-03
Igfbp4	-1.70	4.43E-03	4.81E-04

Cyb5a	-1.70	4.07E-03	4.31E-04
Os9	-1.70	4.78E-03	5.26E-04
Slc37a4	-1.71	5.32E-03	6.07E-04
Hmox1	-1.71	2.58E-02	4.35E-03
Cfb	-1.71	1.11E-02	1.51E-03
Dcn	-1.71	1.48E-02	2.18E-03
Haao	-1.72	1.06E-02	1.43E-03
C3	-1.72	2.25E-02	3.67E-03
Mtss1	-1.72	7.42E-03	9.24E-04
Aass	-1.73	3.93E-03	4.12E-04
Aqp1	-1.73	3.63E-03	3.75E-04
P4hb	-1.74	6.34E-03	7.51E-04
Tln1	-1.74	2.63E-02	4.46E-03
Baat	-1.74	2.42E-03	2.27E-04
Ugt2b36	-1.75	1.64E-03	1.40E-04
Crp	-1.75	2.64E-03	2.55E-04
Hbb-b1	-1.75	3.67E-02	6.81E-03
Abhd14b	-1.75	1.41E-03	1.16E-04
Hbb-bs	-1.76	3.57E-02	6.58E-03
Cfh	-1.76	1.19E-02	1.64E-03
Aldh1a1	-1.77	3.80E-03	3.95E-04
Tmem176a	-1.78	5.01E-03	5.61E-04
Slc6a13	-1.78	3.91E-03	4.08E-04
Xdh	-1.78	2.52E-03	2.41E-04
Ghr	-1.80	2.20E-03	2.02E-04
Apoa5	-1.84	8.61E-03	1.11E-03
Gamt	-1.84	1.83E-03	1.59E-04
Slc16a7	-1.85	6.77E-03	8.16E-04
Alas1	-1.85	2.54E-03	2.44E-04
Slc16a2	-1.85	1.13E-03	8.78E-05
Agxt2	-1.85	5.48E-04	3.63E-05
Igf1	-1.85	2.78E-03	2.72E-04
Sult2a8	-1.85	1.37E-02	1.97E-03
Cyp2d9	-1.86	1.20E-02	1.66E-03
Nr1i3	-1.86	8.31E-03	1.06E-03
Ttpa	-1.86	4.63E-03	5.06E-04
Clu	-1.86	3.85E-03	4.02E-04
Slc26a1	-1.87	8.25E-04	5.99E-05
Glyat	-1.87	1.06E-03	8.06E-05
Pipox	-1.88	2.18E-04	1.21E-05
Serpina1b	-1.89	7.97E-04	5.72E-05

Hp	-1.89	5.44E-03	6.25E-04
Gck	-1.90	7.23E-04	5.11E-05
Serpina10	-1.90	2.20E-04	1.23E-05
Ifitm3	-1.91	1.25E-03	9.89E-05
Man2b2	-1.91	7.20E-03	8.84E-04
Fetub	-1.92	1.81E-03	1.57E-04
Cyp2d26	-1.93	1.13E-03	8.79E-05
Tmem176b	-1.93	1.82E-03	1.59E-04
Tomm40l	-1.94	3.98E-03	4.17E-04
Ifitm2	-1.94	1.86E-03	1.63E-04
Rarres2	-1.94	5.70E-04	3.85E-05
Pah	-1.95	6.35E-04	4.35E-05
Ugp2	-1.95	2.73E-04	1.58E-05
Acsm1	-1.95	3.85E-04	2.34E-05
Serpina3n	-1.97	5.28E-04	3.47E-05
Slc9a3r1	-1.98	4.92E-04	3.16E-05
C4b	-1.98	1.40E-03	1.14E-04
Ly6e	-1.98	4.62E-03	5.03E-04
Hc	-1.99	3.21E-04	1.92E-05
Gnmt	-1.99	3.55E-03	3.64E-04
Scarb1	-1.99	7.03E-04	4.93E-05
Actb	-2.01	1.90E-03	1.67E-04
Asl	-2.02	8.27E-04	6.04E-05
Ctsa	-2.04	8.10E-04	5.84E-05
C8g	-2.04	5.60E-04	3.76E-05
Serpina1a	-2.04	6.39E-04	4.41E-05
Lcp1	-2.06	9.84E-04	7.38E-05
Acp5	-2.07	6.11E-04	4.18E-05
Etnk2	-2.07	4.09E-04	2.54E-05
Hpx	-2.08	9.17E-04	6.82E-05
Msn	-2.09	2.45E-03	2.31E-04
Acat3	-2.09	5.87E-05	2.42E-06
Gm4788	-2.10	1.99E-03	1.79E-04
Cyp2c70	-2.11	1.53E-03	1.27E-04
Slco2b1	-2.11	6.20E-05	2.62E-06
Sirpa	-2.12	4.02E-03	4.24E-04
BC024386	-2.13	1.62E-04	8.48E-06
Cdo1	-2.14	6.22E-05	2.64E-06
Trf	-2.14	7.08E-04	4.97E-05
Apcs	-2.15	5.20E-05	2.11E-06
Ece1	-2.16	5.20E-05	2.11E-06

Gfra1	-2.20	3.78E-04	2.28E-05
Serping1	-2.20	7.34E-05	3.20E-06
Rnase4	-2.21	9.90E-05	4.67E-06
Dhrs1	-2.23	1.05E-04	5.01E-06
Sdc3	-2.26	5.46E-04	3.61E-05
Apol7a	-2.29	1.02E-04	4.83E-06
Bgn	-2.29	1.07E-03	8.18E-05
Cyp2a12	-2.29	3.78E-05	1.44E-06
Cfi	-2.29	4.76E-05	1.90E-06
Cyp2e1	-2.30	1.59E-04	8.31E-06
Sord	-2.30	4.88E-05	1.97E-06
Cyp4v3	-2.32	1.28E-05	3.77E-07
Dio1	-2.34	1.51E-02	2.23E-03
Serpina3m	-2.36	2.18E-04	1.21E-05
Flna	-2.37	2.50E-03	2.37E-04
Mug2	-2.38	4.75E-05	1.88E-06
Serpina1d	-2.38	7.52E-05	3.35E-06
Fmo5	-2.39	1.98E-05	6.40E-07
Gpt2	-2.39	2.10E-05	6.86E-07
1810058I24Rik	-2.40	2.00E-04	1.09E-05
Ppp1r3b	-2.44	9.90E-05	4.68E-06
Ces3a	-2.51	4.14E-05	1.61E-06
Fgl1	-2.54	1.67E-05	5.28E-07
Lgmn	-2.55	8.08E-05	3.63E-06
Hsd3b3	-2.57	8.78E-04	6.46E-05
Osgin1	-2.57	2.91E-02	5.06E-03
Csf1r	-2.58	7.05E-05	3.05E-06
Amy1	-2.58	1.04E-05	2.88E-07
Tmsb4x	-2.58	1.42E-04	7.26E-06
Ctsc	-2.59	1.08E-05	3.04E-07
C1qa	-2.67	4.12E-05	1.59E-06
Tsku	-2.71	5.55E-04	3.70E-05
Tgfbi	-2.72	1.17E-04	5.68E-06
Nat8f2	-2.72	3.30E-06	7.36E-08
C1qc	-2.73	1.57E-05	4.86E-07
Slco1b2	-2.77	3.36E-05	1.23E-06
Pon1	-2.83	3.20E-06	6.99E-08
Mpeg1	-2.84	1.20E-05	3.47E-07
Sardh	-2.88	7.24E-07	1.21E-08
C1ra	-2.90	7.24E-07	1.22E-08
Cd5l	-2.90	6.71E-06	1.71E-07

Serpina6	-2.93	1.61E-06	3.07E-08
Cyp2c23	-2.99	2.56E-05	8.64E-07
F5	-3.02	8.76E-06	2.32E-07
C1qb	-3.04	1.50E-05	4.56E-07
H2-Aa	-3.07	1.09E-03	8.39E-05
Spp1	-3.07	9.32E-04	6.95E-05
Serpina3k	-3.08	6.70E-06	1.70E-07
Plvap	-3.10	9.54E-06	2.62E-07
Cyp2f2	-3.10	2.42E-06	5.08E-08
Col3a1	-3.11	1.40E-03	1.15E-04
Prelp	-3.15	3.18E-05	1.15E-06
Bhmt	-3.22	2.37E-06	4.96E-08
Laptm5	-3.23	3.34E-05	1.22E-06
H2-Eb1	-3.24	1.67E-03	1.44E-04
Mug1	-3.25	4.01E-06	9.34E-08
H2-Ab1	-3.31	1.07E-03	8.18E-05
Cd74	-3.33	1.75E-03	1.51E-04
Mup7	-3.37	6.32E-03	7.46E-04
Glul	-3.48	4.18E-07	6.11E-09
Otc	-3.50	1.06E-05	2.95E-07
Cyp2c67	-3.51	1.00E-07	1.18E-09
Lyz2	-3.70	1.88E-05	6.04E-07
Cyp3a11	-3.72	2.37E-06	4.94E-08
Ctss	-3.81	1.34E-06	2.45E-08
Ccl6	-3.82	3.45E-06	7.87E-08
Rorc	-3.85	1.47E-05	4.41E-07
Itih4	-3.98	6.96E-08	7.71E-10
Sult1a1	-4.03	2.65E-07	3.58E-09
C4bp	-4.16	4.14E-09	2.83E-11
Cyp2c29	-4.21	3.45E-06	7.84E-08
Gpld1	-4.23	1.88E-08	1.72E-10
Cyp1a2	-4.29	1.70E-07	2.11E-09
Akr1c6	-4.34	8.18E-09	6.23E-11
Hrg	-4.38	1.38E-08	1.18E-10
Ces1b	-4.47	8.31E-09	6.40E-11
Ces1c	-4.58	3.14E-09	1.98E-11
Lrg1	-4.59	7.17E-07	1.17E-08
Steap4	-4.62	4.30E-08	4.44E-10
Itgb2	-4.91	7.89E-07	1.38E-08
Serpina1c	-4.93	1.29E-08	1.09E-10
C1s1	-4.94	1.21E-09	6.41E-12

Cyp2a5	-5.01	2.18E-08	2.03E-10
Orm1	-5.13	1.84E-08	1.67E-10
Mbl2	-5.88	7.17E-07	1.18E-08
Plin4	-6.09	4.36E-07	6.45E-09
Lect2	-6.85	6.13E-10	2.60E-12
Gulo	-7.00	6.97E-10	3.44E-12
Saa4	-7.07	5.04E-11	1.25E-13
Prodh	-7.26	6.23E-11	1.73E-13
Aox3	-7.77	6.55E-10	3.02E-12
Enpp2	-7.78	4.31E-09	2.98E-11
C8a	-10.43	2.17E-10	7.69E-13
C9	-10.43	3.63E-09	2.42E-11
Mup20	-11.67	5.04E-10	2.09E-12
Ugt2b1	-13.20	8.64E-12	1.79E-14
Hsd11b1	-15.99	7.96E-15	6.20E-18
Car3	-16.63	5.57E-11	1.45E-13
Oat	-23.05	2.47E-14	2.56E-17
Lcn2	-25.23	5.87E-09	4.32E-11
Saa1	-37.33	8.90E-15	7.70E-18
Mup3	-41.13	9.66E-13	1.42E-15
Apoa4	-44.16	2.17E-10	7.68E-13
Cyp2c54	-51.56	1.19E-08	9.54E-11
Serpina1e	-64.45	2.08E-10	6.83E-13
Pigr	-99.94	1.38E-18	1.20E-22
Cyp2c37	-156.46	1.24E-15	3.22E-19
Cyp2c50	-193.05	3.42E-05	1.26E-06
